# Associations between temporomandibular disorders/bruxism and head and neck pains: a bidirectional Mendelian randomization study

**DOI:** 10.22514/jofph.2025.070

**Published:** 2025-12-12

**Authors:** Shan-Shan Du, Yuan-Yuan Hu, Yu-Ming Niu

**Affiliations:** ^1^Department of Stomatology, Taihe Hospital, Hubei University of Medicine, 442000 Shiyan, Hubei, China; ^2^Department of Stomatology, Gongli Hospital of Shanghai Pudong New Area, 200135 Shanghai, China; ^3^Hubei Key Laboratory of Embryonic Stem Cell Research, Hubei Provincial Clinical Research Center for Umbilical Cord Blood Hematopoietic Stem Cells, Taihe Hospital, Hubei University of Medicine, 442000 Shiyan, Hubei, China

**Keywords:** Temporomandibular disorders, Bruxism, Headache, Pain, Mendelian randomization

## Abstract

**Background**: Temporomandibular disorders (TMDs) and bruxism are the most common disorders affecting the oral and maxillofacial system. Patients with TMDs or bruxism frequently suffer from chronic head and neck pains (HNPs); however, the etiology between TMDs/bruxism and HNPs remains unclear. **Methods**: We explore the association between TMDs/bruxism and HNPs with a bidirectional Mendelian randomization (MR) method with public online genome-wide association study (GWAS) data from the Integrative Epidemiology Unit (IEU) open GWAS project, FinnGen consortium and GWAS Catalog website. Inverse variance weighted (IVW) and other four statistical approaches were employed to investigate the associations. Furthermore, Cochran’s Q, Mendelian randomization (MR)-Egger intercept test, MR pleiotropy residual sum and outlier test, and leave-one-out tests were conducted as sensitivity analyses to ensure the robustness of results. Multivariable MR (MVMR) analyses were adopted to validate the significant effects observed in the two-sample MR analyses while adjusting for potential confounders. **Results**: Overall, our analysis revealed a reciprocal significant association between TMDs and neck/shoulder pain (NSP) in both forward (*p* = 0.023) and reverse (*p* = 0.004) analyses, which were corroborated by subsequent MVMR analyses adjusted for anxiety, body mass index (BMI) and sleeplessness. All results were confirmed robust under current sensitivity analysis. **Conclusions**: These findings suggest a potential causal association between TMDs and HNPs. The bidirectional relationship highlight the importance of preventing TMDs and HNPs as a health strategy for mitigating each other’s risks.

## 1. Introduction

Head and neck pains (HNPs) encompass a range of chronic primary pain disorders, including headaches, migraines, and neck pain, which are the most prevalent nervous system disorders globally [1]. HNPs represent a significant global public health concern and were identified as one of the leading causes of disability in 2019 based on age-standardized disability adjusted life years (DALYs) [2, 3]. Epidemiological studies indicate that most of the general population will experience at least one type HNP during their lifetime, with the highest prevalence observed among young and middle-aged women [4]. Beyond the high frequency, HNPs impose substantial socioeconomic and psychological burdens, and are associated with increased risk of comorbidities, including anxiety [5], depression [6], and suicide ideation [7], particularly among individuals with migraines. Although recent studies have suggested that the dysfunction of the trigeminovascular system, neurogenic inflammation, and various risk factors [8], including poor posture, sleeplessness, chronic stress, obesity, microbiome and vagus, contribute to the development of HNPs, their precise etiology remains uncertain [9].

The oral and maxillofacial system is one of the most functionally complex systems of the human body [10]. Comprising multiple irregularly shaped bones, a bilateral temporomandibular joint (TMJ), and an intricate network of muscles, it facilitates essential functions such as facial expression, speech, respiration, and mastication. Temporomandibular disorders (TMDs) are a group of clinical disorders that affect the TMJ, masticatory muscles, and adjacent structures, and typically accompany with the following symptoms: (1) face and preauricular pain, (2) TMJ sounds (*e.g.*, clicking or popping), and (3) restricted or deviated mandibular movement. In adults, the incidence rate of TMDs is estimated to be 31%, especially higher in women [11]. Despite extensive research, the pathophysiological mechanisms underlying TMDs are not yet fully understood. Current evidence suggests that the etiology is multifactorial, including biomechanics, anatomy, genetics, and psychological factors, and further investigation is therefore required to elucidate their precise pathogenesis [12].

Bruxism is a common orofacial condition characterized by recurrent masticatory muscle activity, such as teeth grindin or clenching, that occurs while awake and asleep [13]. This parafunctional behavior may impair the load-bearing capacity of the TMJ and contribute to the development of TMDs. Recent epidemiological studies report a global prevalence of 22.2% for bruxism, encompassing both sleep and awake forms [14].

According to current evidence, TMDs/bruxism are caused by a multifactorial interplay involving anatomical, biological, environmental, societal, and psychological elements, and are frequently associated with altered occlusal function. A recent study suggested that occlusion can stimulate various regions of the cerebral cortex and may play an important role in the onset and progression of diseases, such as anxiety, stress, and headaches [15]. Moreover, numerous studies have demonstrated that individuals with TMDs or bruxism commonly present with comorbidities, including headaches, neck and back pain, and a variety of social and psychiatric conditions [16].

Mendelian randomization (MR) is a novel epidemiological approach that utilizes single nucleotide polymorphisms (SNPs) as instrumental variables (IVs) to estimate the causal effects of different exposures on health outcomes [17]. MR analysis effectively minimize confounding factors, measurement errors, and reverse causation biases (that frequently undermine the validity of conventional observational studies), thereby significantly enhancing causal inference in epidemiological research [18].

In this study, we hope to elucidate the potential association between TMDs/bruxism and six HNPs types: headache, neck/shoulder pain (NSP), facial pain (FP), cluster headache (CLH), tension-type headache (TTH) and migraine. The study results could provide crucial insights into the shared pathophysiology of these conditions and offer evidence-based foundations for effective interventions.

## 2. Materials and methods

### 2.1 Study design and data sources

A bidirectional two-sample MR analysis was conducted to clarify the exact associtaions between TMDs/bruxism and six HNPs subtypes. For each MR analysis, the selected genetic IVs must meet the following three core assumptions: (1) IVs are strongly related to exposure; (2) IVs are not related to confounding factors; (3) IVs affect the results only through the exposure, rather than through other means. The procedure of this study was illustrated in Fig. 1.

**Fig. 1.** Flowchart of the study design. The red dotted line represented the forward MR analyses, with temporomandibular disorders or bruxism as exposure and head and neck pains as the outcome. The blue dotted line represented the reverse MR analyses, with head and neck pains as exposure and temporomandibular disorders or bruxism as the outcome. MR: Mendelian randomization; IVW: Inverse variance weighted; SNPs: Single-nucleotide polymorphisms; BMI: body mass index; LD: Linkage Disequilibrium; PRESSO: Pleiotropy RESidual Sum and Outlier test.

All pooled data were obtained from the publicly available genome-wide association study (GWAS), and no additional ethical approval was required.

For TMDs, bruxism, and CLH, the data were retrieved from version 11 of the FinnGen Consortium (https://r11.finngen.fi/). Data for headache, NSP, FP, and migraine were obtained from the Integrative Epidemiology Unit (IEU) Open GWAS database (https://gwas.mrcieu.ac.uk/), while the data for TTH were obtained from the GWAS Catalog. In order to minimize population stratification bias, all data were restricted to European individuals. Detailed descriptions of each GWAS datasets were presented in **Supplementary Table 1**. In addition, multivariable Mendelian randomization (MVMR) analyses were conducted to adjust for potential confounders including anxiety, body mass index (BMI), and sleeplessness (https://gwas.mrcieu.ac.uk/), only when statistically significant associations (*p* < 0.05) were identified in the initial univariable MR analysis.

### 2.2 Instrumental variables selection

In the univariable two-sample MR analyses, genetic SNPs were selected as IVs based on a genome-wide significance threshold of *p* < 5 × 10^−8^. Due to the limited number of eligible IVs at this threshold, a relaxed criterion of *p* < 5 × 10^−6^ was additionally applied to enhance the availability of genetic instruments. Any SNPs with linkage disequilibrium, including *r*^2^ < 0.001 and clumping window >10,000 kb was excluded to ensure independence with 1000 Genomes Project. Confounding SNPs were further removed by cross-referencing secondary phenotypes in the LDtrait database (https://ldlink.nih.gov/?tab=ldtrait). Potential weak IV bias were evaluated with *F*-statistic according to the formulas *F* = (beta/Standard Error)^2^ and *R*^2^ = beta^2^/(beta^2^ + N × Standard Error^2^). Any SNP with an *F*-value less than 10 was removed due to a high likelihood of weak instrument bias.

### 2.3 Statistical analysis

In this MR study, the inverse variance-weighted (IVW) method was employed as the primary analytical approach to evaluate the causal link between exposures and outcomes. Cochran’s Q-test was used to assess heterogeneity among the IVs. A fixed-effects model was applied when *p * ≥ 0.05, whereas a random-effects IVW model was adopted if otherwise. To further validate the findings, supplementary MR analyses were conducted using MR-Egger regression, weighted median, simple mode, and weighted mode methods. The intercept term from MR-Egger regression was analyzed for horizontal pleiotropy. A significant intercept (*p* < 0.05) indicated the presence of pleiotropic effects. Furthermore, the MR-Pleiotropy Residual Sum and Outlier (PRESSO) test was implemented to identify and account for any horizontal pleiotropic outliers. Leave-one-out sensitivity analyses were conducted by sequentially excluding individual SNPs to assess the robustness of causal estimates. Furthermore, the MRlap approach was employed to prevent and eliminate the potential bias from sample overlap between TMDs, bruxism, and CLH, as all were sourced from the FinnGen consortium. All statistical analyses were carried out with R software (version 4.3.3), primarily tilizing the “TwoSampleMR” and “MRPRESSO” packages to ensure rigorous and reproducible results.

## 3. Results

### 3.1 TMDs and HNPs

In the forward analysis, 18, 17, 11, 18, 17 and 18 IVs were selected for assessing the causal effects of TMDs on headache, NSP, FP, CLH, TTH, and migraine, respectively. In the reverse analysis, 6, 62, 16, 15, 8 and 57 IVs were selected for assessing the causal effects of these HNPs on TMDs analysis, respectively. All selected IVs had a higher *F* statistical value than 10, and the comprehensive information was presented in **Supplementary Tables 2,3,4,5,6,7,8,9,10,11,12,13**.

According to Cochrane’s Q statistic, significant heterogeneity was identified only in the reverse analysis examining the effect of NSP on TMDs (IVW: Pheterogeneity <0.001), necessitating the use of a random-effects IVW model (Fig. 2). No substantial heterogeneity was detected in the other MR analyses using either IVW or MR-Egger methods between TMDs and other HNPs types (Table 1).

**Fig. 2.** A funnel plot was applied to detect whether the observed association of temporomandibular disorders with neck/shoulder pain was along with obvious heterogeneity. MR: Mendelian randomization; SE: Standard Error; IV: Instrumental Variable.

**Table 1.** Heterogeneity test and horizontal pleiotropy test of temporomandibular disorders and head and neck pains.

Exposure/Outcome	Heterogeneity test (IVW)			Heterogeneity test (MR-Egger)			Horizontal pleiotropy test (MR-Egger)			MR-PRESSO Global test
	Q	*df*	*p*-value	Q	*df*	*p*-value	Intercept	SE	*p*-value	*p*-value
TMDs on headache	5.994958	17	0.9932195	5.994805	16	0.9881515	1.530322 × 10^−6^	0.0001237605	0.9902871	0.995
TMDs on neck/shoulder pain	19.372331	16	0.2498288	9.385965	15	0.8564877	0.001845168	0.0005838915	0.006470998	0.262
TMDs on facial pain	7.076937	10	0.7181622	6.150034	9	0.7248134	−0.0002680206	0.0002783884	0.3608216	0.759
TMDs on cluster headache	24.07403	17	0.1174373	21.85083	16	0.1480738	0.0423796	0.03321549	0.2202035	0.103
TMDs on tension-types headache	5.054717	16	0.9954746	4.731316	15	0.9941672	−0.03018997	0.05308745	0.5779865	0.997
TMDs on migraine	20.14319	17	0.2669709	20.07038	16	0.2170674	0.000251774	−6.065839 × 10^−5^	0.8126741	0.275
Headache on TMDs	3.799261	5	0.5786641	3.761616	4	0.4392277	0.009099361	0.04689826	0.8556125	0.699
Neck/shoulder pain on TMDs	109.0833	61	0.0001526664	109.0637	60	0.0001112949	−0.001515319	0.01461441	0.9177638	0.687
Facial pain on TMDs	10.158033	15	0.8096831	9.414916	14	0.8035885	0.02174379	0.02522356	0.4031886	0.838
Cluster headache on TMDs	18.16753	14	0.1992567	18.09838	13	0.1538195	0.003553817	0.01594575	0.8271009	0.239
Tension-types headache on TMDs	4.409164	7	0.7316261	2.568919	6	0.8606762	−0.03525023	0.02598509	0.223741	0.781
Migraine on TMDs	52.65220	56	0.6023771	52.63471	55	0.5655238	0.00105328	0.00796417	0.8952674	0.579

*TMDs: Temporomandibular disorders; IVW: Inverse variance weighted; MR: Mendelian randomization; PRESSO: Pleiotropy RESidual Sum and Outlier test; df: degrees of freedom; SE: Standard Error.*

Overall, the analysis indicated a significant association between genetically predicted TMDs and NSP (Odds Ratio (OR) = 1.005, 95% Confidence Interval (CI) = 1.001–1.010, *p* = 0.023) (Figs. 3,4, Table 2), and FP (OR = 1.002, 95% CI = 1.000–1.004, *p* = 0.014) in the forward analysis. Interestingly, a reciprocal positive effect of NSP on TMDs (OR = 7.281, 95% CI = 1.900–27.896, *p* = 0.004) was found in the reverse analysis. Moreover, no statistically significant associations was observed between TMDs and other types of HNPs (Table 2). Importantly, no significant pleiotropy bias was observed in either horizontal pleiotropy test (MR-Egger) or the MR-PRESSO global test (Table 1), suggesting that the selected IVs did not exert their effects through alternative biological pathways. Furthermore, the leave-one-out analysis further confirmed the stability of the findings, as the exclusion of individual SNPs did not substantially alter the results, indicating robustness and reliability of the estimates (Fig. 5 for TMDs on NSP analysis).

**Fig. 3.** A forest plot of the association of temporomandibular disorders with neck/shoulder pain. MR: Mendelian randomization.

**Fig. 4.** A scatter plot of the association of temporomandibular disorders with neck/shoulder pain. MR: Mendelian randomization; SNP: single nucleotide polymorphisms.

**Table 2.** Causal association between temporomandibular disorders and head and neck pains.

Exposure/Outcome	SNPs	Methods	OR	95% CI	*p*-Value
TMDs on headache					
	18	MR-Egger	1.001	0.999–1.002	0.426
		Weighted median	1.000	0.999–1.002	0.518
		Inverse variance weighted	1.001	0.999–1.001	0.156
		Simple mode	1.001	0.999–1.002	0.552
		Weighted mode	1.001	0.999–1.002	0.529
TMDs on neck/shoulder pain					
	17	MR-Egger	0.996	0.989–1.003	0.305
		Weighted median	1.000	0.993–1.006	0.905
		Inverse variance weighted	1.005	1.001–1.010	0.023
		Simple mode	0.999	0.988–1.009	0.834
		Weighted mode	0.999	0.992–1.006	0.810
TMDs on facial pain					
	11	MR-Egger	1.004	1.000–1.009	0.090
		Weighted median	1.002	1.000–1.005	0.059
		Inverse variance weighted	1.002	1.000–1.004	0.014
		Simple mode	1.002	0.998–1.006	0.346
		Weighted mode	1.002	0.999–1.006	0.219
TMDs on cluster headache					
	18	MR-Egger	0.690	0.428–1.113	0.148
		Weighted median	0.857	0.627–1.171	0.333
		Inverse variance weighted	0.901	0.701–1.158	0.414
		Simple mode	0.668	0.344–1.297	0.250
		Weighted mode	0.687	0.348–1.355	0.294
TMDs on tension-type headache					
	17	MR-Egger	0.8040864	0.428–1.510	0.508
		Weighted median	0.8135216	0.471–1.406	0.460
		Inverse variance weighted	0.6940272	0.478–1.008	0.055
		Simple mode	0.8376861	0.382–1.838	0.665
		Weighted mode	0.8427518	0.413–1.721	0.645
TMDs on migraine					
	18	MR-Egger	1.000	0.997–1.003	0.780
		Weighted median	1.001	0.998–1.003	0.587
		Inverse variance weighted	0.999	0.998–1.001	0.397
		Simple mode	1.001	0.997–1.005	0.695
		Weighted mode	1.001	0.998–1.004	0.429
Headache on TMDs					
	6	MR-Egger	<0.001	2.147 × 10^−25^–6.597 × 10^17^	0.768
		Weighted median	0.134	7.701 × 10^−10^–2.328 × 10^17^	0.835
		Inverse variance weighted	0.037	6.279 × 10^−9^–2.187 × 10^5^	0.679
		Simple mode	0.175	4.312 × 10^−12^–7.095 × 10^9^	0.894
		Weighted mode	0.087	6.296 × 10^−11^–1.198 × 10^8^	0.829
Neck/shoulder pain on TMDs					
	62	MR-Egger	9.706	0.036–2628.170	0.430
		Weighted median	4.898	1.062–22.593	0.042
		Inverse variance weighted	7.281	1.900–27.896	0.004
		Simple mode	4.086	0.138–121.074	0.419
		Weighted mode	3.397	0.144–80.149	0.451
Facial pain on TMDs					
	16	MR-Egger	<0.001	4.223 × 10^−19^–1.291 × 10^6^	0.343
		Weighted median	0.475	7.041 × 10^−5^–3.202 × 10^3^	0.868
		Inverse variance weighted	0.129	2.087 × 10^−4^–7.988 × 10^1^	0.532
		Simple mode	1.012	1.154 × 10^−6^–9.017 × 10^5^	0.998
		Weighted mode	0.557	1.033 × 10^−6^–3.002 × 10^5^	0.932
Cluster headache on TMDs					
	15	MR-Egger	0.997	0.924–1.075	0.930
		Weighted median	1.004	0.947–1.065	0.883
		Inverse variance weighted	1.003	0.958–1.051	0.894
		Simple mode	1.040	0.958–1.129	0.369
		Weighted mode	1.010	0.939–1.086	0.797
Tension-type headache on TMDs					
	8	MR-Egger	1.105575	1.002–1.220	0.093
		Weighted median	1.019415	0.969–1.072	0.455
		Inverse variance weighted	1.038452	0.999–1.080	0.058
		Simple mode	1.019993	0.959–1.085	0.552
		Weighted mode	1.018602	0.963–1.077	0.539
Migraine on TMDs					
	57	MR-Egger	1.897	0.003–1073.379	0.844
		Weighted median	2.324	0.067–80.561	0.641
		Inverse variance weighted	2.833	0.311–25.832	0.356
		Simple mode	8.577	0.007–9966.082	0.553
		Weighted mode	2.544	0.003–2157.830	0.787

*TMDs: Temporomandibular disorders; OR: Odds ratio; SNPs: Single-nucleotide polymorphisms; CI: Confidence Interval.*

**Fig. 5.** A forest plot of the “leave-one-out” sensitivity analysis method to show the influence of individual SNPs on the result of temporomandibular disorders’ association with neck/shoulder pain. MR: Mendelian randomization.

To assess potential bias due to sample overlap in the TMDs and CLH datasets, the MRlap software package was applied with the following parameters: MR_threshold = 5 × 10^−6^ and MR_pruning_LD = 0.05. The statistical results revealed no significant evidence of overlap bias, with *p*_difference values of 0.173 and 0.794, respectively.

MVMR analyses were conducted for associations that were significant in the primary univariable analyses to determine the direct effects of TMDs and HNPs while adjusting for potential confounders. The MVMR results showed that the association between TMDs and NSP remained significant in both forward (OR = 1.019, 95% CI = 1.009–1.029, *p* < 0.001) and reverse analyses (OR = 13.909, 95% CI = 2.033–95.176, *p* = 0.007), after adjustment for anxiety, BMI, and sleeplessness (Table 1).

### 3.2 Bruxism and HNPs

In the forward MR analysis, 9, 10, 6, 11, 10 and 9 IVs were used to assess the causal effects of bruxism on headache, NSP, FP, CLH, TTH, and migraine, respectively. In the reverse analysis, 6, 62, 16, 15, 8 and 57 IVs were used to investigate the effects of these HNP types on bruxism. All selected IVs had a higher *F* statistical value than 10, and the comprehensive information was presented in **Supplementary Tables 14,15,16,17,18,19,20,21,22,23,24,25**.

According to Cochran’s Q statistic, no significant heterogeneity was observed in any analysis on the effect of bruxism and HNPs types in both forward and reverse analysis (Table 3). Overall, the current analysis indicated that there was no significant association between bruxism and any of the six HNPs types (Table 4). Moreover, no significant pleiotropy bias was detected in either the horizontal pleiotropy test (MR-Egger) or the MR-PRESSO global test (Table 3), suggesting that the selected IVs are unlikely to affect the results through alternative causal pathways.

**Table 3.** Heterogeneity test and horizontal pleiotropy test of bruxism and head and neck pains.

Exposure/Outcome	Heterogeneity test (IVW)			Heterogeneity test (MR-Egger)			Horizontal pleiotropy test (MR-Egger)			MR-PRESSO Global test
	Q	*df*	*p*-value	Q	*df*	*p*-value	Intercept	SE	*p*-value	*p*-value
Bruxism on headache	5.845026	8	0.6645863	5.626580	7	0.5839630	−7.460302 × 10^−5^	0.0001596189	0.6544269	0.702
Bruxism on neck/shoulder pain	10.709295	9	0.2961627	7.477352	8	0.4861107	0.001281215	0.0007126725	0.1099286	0.392
Bruxism on facial pain	0.9597556	5	0.9657460	0.7254465	4	0.9481542	−0.0001135888	0.0002346611	0.6536549	0.939
Bruxism on cluster headache	5.082503	10	0.8855989	4.503220	9	0.8752896	0.02848001	0.03741923	0.4660667	0.912
Bruxism on tension-type headache	11.28750	8	0.1859351	11.84828	9	0.2219981	0.0518246	0.08220378	0.5459893	0.267
Bruxism on migraine	9.807297	8	0.1415298	12.222958	7	0.1997593	−0.0004506363	0.0003431893	0.2305594	0.256
Headache on bruxism	1.413464	5	0.9228348	1.400343	4	0.8441354	0.01814381	0.1583946	0.9143229	0.939
Neck/shoulder pain on bruxism	65.09826	61	0.3361056	62.69980	60	0.3807406	−0.05660124	0.03736089	0.1350265	0.339
Facial pain on bruxism	19.84210	15	0.1780740	19.80621	14	0.1363705	0.01618084	0.1015928	0.8757299	0.166
Cluster headache on Bruxism	9.754829	14	0.7798796	8.461027	13	0.8123234	0.04669183	0.04104942	0.2758794	0.589
Tension-type headache on bruxism	4.943647	7	0.6668402	4.537073	6	0.6043991	0.05660817	0.08877875	0.5472595	0.728
Migraine on bruxism	66.02237	56	0.1690467	65.12310	55	0.1649476	−0.02566901	0.02945443	0.3872793	0.177

*TMDs: Temporomandibular disorders; IVW: Inverse variance weighted; MR: Mendelian randomization; PRESSO: Pleiotropy RESidual Sum and Outlier test; df: degrees of freedom; SE: Standard Error.*

**Table 4.** Causal association between of bruxism and head and neck pains.

Exposure/Outcome	SNPs	Methods	OR	95% CI	*p*-Value
Bruxism on headache					
	9	MR-Egger	1.000	1.000–1.001	0.490
		Weighted median	1.000	1.000–1.001	0.403
		Inverse variance weighted	1.000	1.000–1.000	0.570
		Simple mode	1.000	1.000–1.001	0.586
		Weighted mode	1.000	1.000–1.001	0.330
Bruxism on neck/shoulder pain					
	10	MR-Egger	0.999	0.997–1.001	0.391
		Weighted median	1.000	0.998–1.002	0.962
		Inverse variance weighted	1.000	0.999–1.002	0.574
		Simple mode	1.000	0.997–1.004	0.877
		Weighted mode	1.000	0.998–1.002	0.992
Bruxism on facial pain					
	6	MR-Egger	1.000	0.999–1.000	0.425
		Weighted median	1.000	0.999–1.000	0.277
		Inverse variance weighted	1.000	0.999–1.000	0.072
		Simple mode	1.000	0.999–1.001	0.524
		Weighted mode	1.000	0.999–1.000	0.293
Bruxism on cluster headache					
	11	MR-Egger	0.961	0.832–1.110	0.604
		Weighted median	1.003	0.907–1.108	0.957
		Inverse variance weighted	1.008	0.932–1.089	0.848
		Simple mode	0.995	0.872–1.135	0.945
		Weighted mode	0.997	0.886–1.122	0.961
Bruxism on tension-type headache					
	10	MR-Egger	0.9766082	0.774–1.232	0.847
		Weighted median	0.9787152	0.822–1.165	0.809
		Inverse variance weighted	1.0314156	0.885–1.202	0.692
		Simple mode	1.0011730	0.696–1.439	0.995
		Weighted mode	0.9597507	0.807–1.141	0.653
Bruxism on migraine					
	9	MR-Egger	1.001	1.000–1.002	0.332
		Weighted median	1.000	0.999–1.001	0.650
		Inverse variance weighted	1.000	0.999–1.001	0.909
		Simple mode	1.001	0.999–1.002	0.266
		Weighted mode	1.000	1.000–1.001	0.546
Headache on bruxism					
	6	MR-Egger	<0.001	7.644 × 10^−86^–6.064 × 10^58^	0.738
		Weighted median	<0.001	7.814 × 10^−36^–1.249 × 10^21^	0.625
		Inverse variance weighted	<0.001	6.937 × 10^−33^–6.230 × 10^13^	0.433
		Simple mode	<0.001	1.375 × 10^−50^–1.628 × 10^24^	0.527
		Weighted mode	<0.001	9.140 × 10^−39^–7.934 × 10^24^	0.699
Neck/shoulder pain on bruxism					
	62	MR-Egger	464,296.225	0.284–7.587 × 10^11^	0.079
		Weighted median	20.345	0.131–3.152 × 10^3^	0.226
		Inverse variance weighted	10.186	0.304–3.411 × 10^2^	0.195
		Simple mode	43.635	0.000–6.338 × 10^6^	0.512
		Weighted mode	10.841	0.000–4.449 × 10^5^	0.633
Facial pain on bruxism					
	16	MR-Egger	<0.001	6.146 × 10^−59^–2.432 × 10^40^	0.728
		Weighted median	887.590	2.988 × 10^−11^–2.636 × 10^16^	0.668
		Inverse variance weighted	<0.001	1.294 × 10^−16^–7.302 × 10^5^	0.366
		Simple mode	898,885,800	5.179 × 10^−15^–1.560 × 10^32^	0.462
		Weighted mode	5,420,380,000	3.007 × 10^−11^–9.771 × 10^29^	0.361
Cluster headache on bruxism					
	15	MR-Egger	1.036	0.835–1.286	0.752
		Weighted median	1.046	0.857–1.276	0.660
		Inverse variance weighted	1.141	0.993–1.311	0.064
		Simple mode	1.375	0.986–1.918	0.081
		Weighted mode	1.022	0.808–1.294	0.866
Tension-type headache on Bruxism					
	8	MR-Egger	1.009	0.720–1.415	0.959
		Weighted median	1.107	0.926–1.323	0.265
		Inverse variance weighted	1.116	0.978–1.275	0.103
		Simple mode	1.092	0.853–1.397	0.507
		Weighted mode	1.124	0.903–1.400	0.330
Migraine on bruxism					
	57	MR-Egger	9.677 × 10^6^	6.172 × 10^−4^–1.517 × 10^17^	0.185
		Weighted median	8.849 × 10^0^	1.203 × 10^−4^–6.511 × 10^5^	0.710
		Inverse variance weighted	5.430 × 10^2^	1.590 × 10^−1^–1.854 × 10^6^	0.129
		Simple mode	1.113 × 10^−1^	5.328 × 10^−12^–2.325 × 10^9^	0.855
		Weighted mode	7.079 × 10^−2^	2.694 × 10^−9^–1.861 × 10^6^	0.772

*SNPs: Single-nucleotide polymorphisms; MR: Mendelian randomization; OR: Odds Ratio; CI: Confidence Interval.*

The leave-one-out sensitivity analyses further supported the robustness of the findings. The sequential exclusion of individual SNPs did not result in substantial changes in the outcomes, indicating that the causal estimates are stable and reliable.

To assess potential bias arising from data overlap between bruxism and CLH datasets, the MRlap software package was employed with the following parameters: MR_threshold = 5 × 10^−6^ and MR_pruning_LD = 0.05. The analysis indicated no evidence of overlap bias, with *p*_difference values of 1.000 and 0.464, respectively.

## 4. Discussion

TMDs comprise a group of conditions affecting the masticatory muscles, TMJs, and associated tissues. Clinical symptoms include discomfort in the TMJ region, joint popping, and restricted mandibular mobility. Epidemiological studies estimate that TMDs affect approximately 31% of adults/elderly and 11% of children/adolescents [5], with a prevalence nearly twice as high in females than males [19]. Notably, a higher proportion of patients with TMDs experience concurrent symptoms such as headache, migraine, TTH, or neck pain, and this co-occurrence of multiregional pain presents challenges for accurate clinical diagnosis and effective treatment [20, 21]. Similarly, bruxism is a prevalent condition involving involuntary masticatory muscle activity during wakefulness and/or sleep. Its estimated occurrence ranges from 4% to 32%, with a higher prevalence among females [22]. Persistent abnormal muscle contractions may result in muscular fatigue, pain, and various clinical symptoms characteristic of TMDs. A recent study reported a global co-occurrence rate of 17% for bruxism and TMDs, with the highest prevalence observed in North America (70%) [23]. TMDs, bruxism, and HNPs are known to interact in complex ways. For instance, Romero-Reyes and Bassiur reported that anxiety and awake bruxism were significant risk factors for frequent episodic TTHs in individuals with painful TMDs, whereas only awake bruxism was implicated in cases of non-painful TMDs [24]. Voß *et al*. [25] also identified correlations between TMD-related pain and migraine, as well as between awake bruxism and TTH, with several psychosocial variables acting as confounding factors in these relationships. The extensive co-morbidity among pain in the head, face, and neck regions and TMDs/bruxism supports the hypothesis of a shared pathophysiological mechanism.

In this MR investigation, we employed a bidirectional MR to evaluate the potential causal associations between TMDs, bruxism, and six subtypes of HNPs with the most latest publicly available GWAS databases. Our results indicated that genetically predicted TMDs are associated with an increased risk of NSP, and *vice versa*. Subsequent MVMR analyses further validated this association after adjusting for anxiety, BMI, and sleeplessness. Although the observed odds ratio (OR = 1.005) for the effect of TMDs on NSP was statistically significant, its proximity to 1.0 highlights the need to distinguish between statistical significance and clinical relevance. Previous studies have established that TMDs and NSP share comorbid features and may be causally linked in their underlying pathogenesis. Given the high prevalence of both conditions in the general population, even modest associations may translate into meaningful public health implications. Consequently, minor reductions in risk may still hold clinical importance. Conversely, the current findings do not support a significant association between bruxism and any HNPs subtypes. Several factors may account for this result. First, phenotypic heterogeneity in both bruxism and HNPs could have attenuated potential associations. Bruxism exhibits circadian variability and comprises multiple subtypes; however, subgroup analyses could not be performed due to limited sample availability. Second, insufficient sample size may have reduced statistical power, increasing the risk of false-negative findings. Third, the GWAS data utilized in this study were exclusively derived from individuals of European ancestry, limiting the generalizability of the findings to other populations. Given the similar pathogenesis and close clinical relevance of TMDs and bruxism, further research is warranted to clarify the relationship between bruxism and HNPs.

NSP is one of the most prevalent chronic pain, and it can arise from acute or chronic cumulative musculoskeletal injuries. Although TMDs and NSP are two prevalent clinical syndromes, their relationship is often underrecognized by clinical practitioners. A recent cross-sectional study reported that patients with coexisting NSP and TMDs had significantly higher scores on both Bournemouth Neck Questionnaire and Neck Disability Index compared to those with NSP only (*p* < 0.001), highlighting a strong correlation between NSP and TMD-related pain [26]. Serkan *et al*. [27] demonstrated that individuals with both TMDs and NSP exhibited increased frequency, stiffness, and decrement values in the masseter and upper trapezius muscles relative to healthy control subjects (*p* < 0.017). Furthermore, adolescents with TMDs showed significantly higher rates of NSP (*p* < 0.001), which was associated with diurnal clenching behavior [28].

The mechanisms underlying the association between TMDs and NSP are complex and involve interactions among neural, muscular, and biomechanical systems. As early as 1998, Eriksson *et al*. [29] demonstrated that neck muscle activity during head-neck movements was synchronized with electromyographic recordings.

The skull, mandible, and cervical spine form an interconnected unit referred to as the “craniocervico-mandibular system”, wherein the masticatory and cervical muscles function as an integrated network [30].

The muscle chains of the stomatognathic system play a critical role in maintaining mandibular position and musculoskeletal balance. Postural deviations, such as anterior head tilt, lead to compensatory curvature of the thoracic and cervical spine, causing abnormal tension in neck-related musculature, thereby contributing to TMJ dysfunction. Individuals with TMDs frequently present with upper airway restriction and adopt forward head posture to optimize pharyngeal space [31, 32], which alters the position of the cervical spine and increases the burden on neck and shoulder muscle groups [33, 34]. Armijo-Olivo reported that TMDs patients often suffer from NSP, tenderness of cervical joints, and reduced range of motion (ROM) in both the upper and entire cervical spine, frequently accompanied by myofascial trigger points. The study also suggested that altered cervical neuromuscular control may stimulate pain-sensitive structures, contributing to or exacerbating discomfort in the neck and oral/facial regions [35]. Yüzbaşıoğlu noted that individuals with TMDs experience more severe TMJ pain, jaw opening limitations, and dysfunction when presenting with forward head posture, which alters muscle activation patterns and affects neck muscle strength, endurance, and position sense. Similarly, individuals with obstructive sleep apnea and Class II malocclusion often exhibit exaggerated cervical curvature [36, 37]. The presence of TMDs or NSP may disrupt the biomechanical integrity of the craniocervical system, prompting compensatory adaptations and resulting in dysfunction and chronic inflammation in surrounding tissues [38].

Botros *et al*. [39] found that patients with chronic TMDs were more than twice as likely to report NSP (OR = 2.17‎, 95% CI = 1.27–3.71) compared with those with acute TMDs. This observation supports the involvement of central dysfunction, including peripheral and central sensitization mechanisms, in the chronicity of TMD-related pain [40]. Moreover, another suggested mechanism is trigeminocervical convergence [41]. Anatomically, the trigeminocervical nucleus contains overlapping inputs from both trigeminal and cervical spinal nerves, facilitating cross-regional pain transmission [42, 43]. The nucleus of the trigeminal spinal tract is tightly linked to the cervical spinal cord. The nucleus of the trigeminal spinal tract extends from the pons to the medulla oblongata and down to the C2 segment of the spinal cord, with partial projections to the gelatinous substance of the C4 and C6 segments, thus contributing to cervical spinal nerve integration [44]. Neurotracing studies have demonstrated strong synaptic connections between the trigeminal nerve and the upper cervical nerve roots at the spinal trigeminal nucleus (Sp5C), providing a structural basis for cross-regional pain transmission [45, 46]. These reflexive interactions between nociceptors and mechanoreceptors of the TMJ and cervical musculature may contribute to sensory-motor dysfunction in the necks of patients with TMDs [47]. In addition, abnormal sensory input from upper cervical spinal neurons, especially the C2 and C3 nerve roots, may impair maxillofacial muscle coordination when these roots are compressed or pathologically misaligned. Such neural interference can result in muscle spasms, HNP, difficulty in mandibular movement, and characteristic TMJ symptoms such as clicking or popping [48, 49].

Furthermore, Hong *et al*. [50] found that individuals with myofascial TMDs and neck pain exhibited a greater number of active trigger points (TrPs), forward head posture, and more severe cervical degenerative changes than those with myofascial TMDs alone. Furthermore, central sensitization is considered a key mechanism underlying TMD-related multiregional pain [47], which causes the central nervous system to become more receptive to nociceptive stimuli, resulting in hyperalgesia. Quantitative sensory testing has shown that patients with TMDs exhibit reduced heat and mechanical pain thresholds even in regions distant from the TMJ, suggesting widespread central sensitization [51, 52]. Costa *et al*. [53] proposed that dysfunction of the descending pain inhibitory system contributes to comorbid conditions such as TMDs and fibromyalgia. These regulatory neurons are located in the midbrain and medulla, such as the periaqueductal gray matter and ventromedial medulla, and play a critical role in modulating nociceptive input, thereby functioning as endogenous analgesics [54]. In chronic pain states, this system becomes dysregulated, leading to amplified pain perception [55]. Research by Yekkalam *et al*. [52] indicates that frequent FP is linked with both localized and widespread hyperalgesia, likely driven by central sensitization mechanisms. The most frequent TrP locations fall within the distribution of the trigeminal nerve and its overlapping cervical branches. These areas are closely associated with sensorimotor function, involving the convergence of afferent inputs to the brainstem and the transmission of nociceptive signals [36, 56]. Central sensitization may also increase the number of TrPs, thereby contributing to more diffuse pain [57]. This mechanism plays a central role in the bidirectional association between TMDs and NSP. Persistent nociceptive input from TMJ injury can initiate central sensitization in the neck and shoulder regions [51], lowering mechanical pain thresholds and prolonging pain response to pressure stimulation [58, 59].

Notably, inflammatory cytokines also play an essential role in the interaction between TMDs and NSP [60]. Patients with TMDs release several pro-inflammatory cytokines, including interleukin-1 beta (IL-1β) and tumor necrosis factor-alpha (TNF-α), which activate trigeminal ganglion neurons and contribute to peripheral sensitization [61, 62]. Similarly, myofascitis of the neck and shoulder muscles up-regulates the levels of IL-1, IL-6 and TNF-α, which can induce central sensitization directly [63]. Furthermore, neuropeptides such as calcitonin gene-related peptide (CGRP), and substance P are also involved in the neurogenic inflammatory response in both illnesses. These mediators initiate and sustain pain, thereby facilitating central sensitization and its persistence [64]. Elevated CGRP levels have been shown to maintain central sensitization and promote pathological pain states by activating astrocytes and prolonging inflammatory responses [65]. A rat model of TMJ synovitis revealed increased expression of substance P and protein gene product 9.5 (PGP9.5) in inflamed synovial tissues, supporting a neural basis for persistent inflammation [66]. These findings suggest novel therapeutic targets for both TMDs and NSP, including non-steroidal anti-inflammatory drugs (NSAIDs), CGRP inhibitors, and acupuncture, all of which may alleviate symptoms effectively [67, 68].

To our acknowledge, this MR study systematically investigated the relationship between TMDs, bruxism, and HNPs. This study offers several advantages. Firstly, the participants were drawn from the most recent and largest available GWAS sample groups, ensuring the relevance and reliability of our results. Secondly, multiple statistical methods were used to verify the heterogeneity and horizontal pleiotropy, with all yielding ideal results. Finally, MVMR analyses were performed to further eliminate the interference of other potential confounding factors on the results, enhancing the validity of the results. However, there were some shortcomings that should be addressed. First and foremost, all GWAS data in this study were derived from individuals of European ancestry. As recent research suggests that the global prevalence of TMDs and bruxism varies by region, the applicability of our findings to other populations is limited. For example, TMDs prevalence is highest in South America (47%), followed by Asia (33%) and Europe (29%) [11]. Bruxism prevalence also differs significantly across continents: North America (31%), South America (23%), Europe (21%), and Asia (19%) [14], which restricting the applicability of the study’s conclusions to other ethnic groups. These findings must, therefore, be validated through further studies involving more diverse populations. Second, a significant heterogeneity was observed in the analysis of TMDs on NSP, which may lead to some bias. Third, although MVMR accounted for confounders such as anxiety, BMI, and sleeplessness, other potential confounding variables were not fully considered, such as obstructive sleep apnea, which was thought to be associated with TMDs [69]. Finally, previous studies have reported that women are more likely to develop TMDs and NSP than men. However, this study did not perform gender-specific subgroup analyses, limiting the interpretation of gender differences.

## 5. Conclusions

In this MR study, we identified a significant bidirectional relationship between TMDs and NSP, which remained robust even after adjustment for potential confounders. In addition to muscular posture chains, central nervous system dysregulation, and inflammation processes, other potential mechanisms such as psychological disorders, occupational environment, and gender differences may influence the pathogenesis of TMDs and NSP. These hypotheses warrant further investigation in future research.
