# Supplementary material for: Associations between temporomandibular disorders/bruxism and head and neck pains: a bidirectional Mendelian randomization study
Source: J Oral Facial Pain Headache. 2025 Dec 12;39(4):122–37. doi: 10.22514/jofph.2025.070 (PMC12727188; doi:10.22514/jofph.2025.070)
Supplement: Supplementary file 1 [file Supplementary-material.docx]

Supplementary material

Supplementary Table 1. Detailed information of the GWAS included in Mendelian randomization analysis.

| Phenocode | GWAS ID | Databases | Sample size | PMID | Web source |
| --- | --- | --- | --- | --- | --- |
| TMDs | finngen_R11_TEMPOROMANDIB  247,966 | FinnGen | 7102/240,864 | NA | <https://storage.googleapis.com/finngen-public-data-r11/summary_stats/finngen_R11_TEMPOROMANDIB.gz> |
| Bruxism | finngen_R11_BRUXISM  453,733 | FinnGen | 591/453,142 | NA | <https://storage.googleapis.com/finngen-public-data-r11/summary_stats/finngen_R11_BRUXISM.gz> |
| Headaches (not migraine) | ebi-a-GCST90038675  484,598 | IEU | 4122/480,476 | 33959723 | <https://www.nature.com/articles/s41588-023-01538-0> |
| Neck/shoulder pain | ukb-b-18596  461,857 | IEU | 106,521/355,336 | NA | <https://www.nature.com/articles/s41588-021-00990-0> |
| Facial pain | ukb-b-17107  461,857 | IEU | 8595/453,262 | NA | <https://www.nature.com/articles/s42003-021-02356-y> |
| Cluster headache | finngen_R11_G6_CLUSTHEADACHE_WIDE  343,239 | FinnGen | 1355/341,884 | NA | <https://storage.googleapis.com/finngen-public-data-r11/summary_stats/finngen_R11_G6_CLUSTHEADACHE_WIDE.gz> |
| Tension-types headache | GCST90435882  365,791 | Catalog GWAS | 315/365,476 | 30104761 | <https://www.ebi.ac.uk/gwas/studies/GCST90435882> |
| Migraine | ebi-a-GCST90038646  484,598 | IEU | 13,971/470,627 | 33959723 | <https://gwas.mrcieu.ac.uk/datasets/ebi-a-GCST90038646/> |

TMDs: Temporomandibular joint disorders; GWAS: genome-wide association study; NA: Not available; IEU: Integrative Epidemiology Unit.

Supplementary Table 2. Detail information of selected SNPs for MR analysis of the causal effect of TMDs on headache.

| SNP | EA | OA | Exposure | | | | | Outcome | | | |
| --- | --- | --- | --- | --- | --- | --- | --- | --- | --- | --- | --- |
|  |  |  | Beta | SE | *p*-value | EAF | *F* | Beta | SE | *p*-value | EAF |
| rs10882591 | T | C | −0.162649 | 0.031081 | 1.67 × 10^−7^ | 0.0909964 | 27.38553 | −0.00020081 | 0.000346 | 0.56 | 0.081128 |
| rs111626487 | A | G | 0.252379 | 0.054841 | 4.18 × 10^−6^ | 0.0222109 | 21.1786 | 0.000170467 | 0.00073 | 0.82 | 0.016796 |
| rs112370138 | T | C | −0.22908 | 0.0482 | 2.01 × 10^−6^ | 0.0380217 | 22.58832 | −8.15 × 10^−6^ | 0.000442 | 0.99 | 0.047239 |
| rs114568702 | C | T | −0.376846 | 0.079973 | 2.45 × 10^−6^ | 0.0151118 | 22.20439 | −0.00015326 | 0.000703 | 0.83 | 0.019867 |
| rs11675477 | C | A | 0.185278 | 0.039844 | 3.32 × 10^−6^ | 0.94562 | 21.62373 | −0.0004289 | 0.00047 | 0.36 | 0.953507 |
| rs137964046 | A | G | −0.688037 | 0.147387 | 3.04 × 10^−6^ | 0.00548901 | 21.79241 | −0.00024737 | 0.001053 | 0.81 | 0.010755 |
| rs139330 | A | C | 0.0842086 | 0.017731 | 2.04 × 10^−6^ | 0.36942 | 22.55519 | −6.51 × 10^−5^ | 0.000216 | 0.760001 | 0.255593 |
| rs1960036 | A | C | 0.0977597 | 0.021291 | 4.40 × 10^−6^ | 0.198771 | 21.08376 | 0.000133665 | 0.000245 | 0.58 | 0.180862 |
| rs199894590 | T | G | 0.106329 | 0.022159 | 1.60 × 10^−6^ | 0.181694 | 23.02581 | −9.37 × 10^−5^ | 0.000315 | 0.77 | 0.123604 |
| rs2058908 | C | T | −0.0863736 | 0.018036 | 1.68 × 10^−6^ | 0.663399 | 22.93359 | −0.00034061 | 0.000214 | 0.11 | 0.741022 |
| rs216497 | G | T | 0.0905763 | 0.019773 | 4.63 × 10^−6^ | 0.732557 | 20.98337 | −2.63 × 10^−5^ | 0.000201 | 0.9 | 0.680593 |
| rs2531358 | G | A | −0.0808625 | 0.017253 | 2.77 × 10^−6^ | 0.534788 | 21.96748 | 7.91 × 10^−65^ | 0.000189 | 0.68 | 0.442773 |
| rs4739605 | T | C | 0.62815 | 0.13225 | 2.04 × 10^−6^ | 0.993498 | 22.55981 | 0.000244662 | 0.000632 | 0.7 | 0.976144 |
| rs58472553 | T | G | 0.16029 | 0.034044 | 2.50 × 10^−6^ | 0.062276 | 22.16852 | 8.09 × 10^−5^ | 0.000391 | 0.84 | 0.060745 |
| rs6752506 | G | A | −0.0990722 | 0.019331 | 2.97 × 10^−7^ | 0.282251 | 26.26661 | −0.00017407 | 0.000217 | 0.42 | 0.252163 |
| rs72994670 | C | T | 0.225847 | 0.046984 | 1.53 × 10^−6^ | 0.0322143 | 23.10581 | 0.000325195 | 0.000424 | 0.44 | 0.05176 |
| rs76537824 | C | T | 0.278102 | 0.060449 | 4.21 × 10^−6^ | 0.018104 | 21.16592 | 0.000533943 | 0.000393 | 0.17 | 0.064006 |
| rs77392397 | G | C | 0.320963 | 0.069124 | 3.43 × 10^−6^ | 0.0138646 | 21.56017 | 0.000239723 | 0.00052 | 0.64 | 0.033522 |

EA: Effect allele; OA: Other allele; EAF: Effect allele frequency; TMDs: Temporomandibular disorders; SNP: Single nucleotide polymorphism; SE: Standard Error.

Supplementary Table 3. Detail information of selected SNPs for MR analysis of the causal effect of TMDs on neck/shoulder pain.

| SNP | EA | OA | Exposure | | | | | Outcome | | | |
| --- | --- | --- | --- | --- | --- | --- | --- | --- | --- | --- | --- |
|  |  |  | Beta | SE | *p*-value | EAF | *F* | Beta | SE | *p*-value | EAF |
| rs10882591 | T | C | −0.16265 | 0.031081 | 1.67 × 10^−7^ | 0.090996 | 27.38553 | 0.000359 | 0.001616 | 0.82 | 0.080914 |
| rs111626487 | A | G | 0.252379 | 0.054841 | 4.18 × 10^−6^ | 0.022211 | 21.1786 | −3.27 × 10^−5^ | 0.003352 | 0.99 | 0.017441 |
| rs112370138 | T | C | −0.22908 | 0.0482 | 2.01 × 10^−6^ | 0.038022 | 22.58832 | 0.001171 | 0.002037 | 0.57 | 0.048757 |
| rs114568702 | C | T | −0.37685 | 0.079973 | 2.45 × 10^−6^ | 0.015112 | 22.20439 | 0.00034 | 0.003227 | 0.92 | 0.020561 |
| rs11675477 | C | A | 0.185278 | 0.039844 | 3.32 × 10^−6^ | 0.94562 | 21.62373 | 0.001911 | 0.002295 | 0.41 | 0.961524 |
| rs137964046 | A | G | −0.68804 | 0.147387 | 3.04 × 10^−6^ | 0.005489 | 21.79241 | −0.00174 | 0.004834 | 0.719999 | 0.011105 |
| rs139330 | A | C | 0.084209 | 0.017731 | 2.04 × 10^−6^ | 0.36942 | 22.55519 | 0.00073 | 0.001003 | 0.47 | 0.258042 |
| rs1960036 | A | C | 0.09776 | 0.021291 | 4.40 × 10^−6^ | 0.198771 | 21.08376 | 0.001351 | 0.001138 | 0.24 | 0.182171 |
| rs2058908 | C | T | −0.08637 | 0.018036 | 1.68 × 10^−6^ | 0.663399 | 22.93359 | −0.00116 | 0.000994 | 0.24 | 0.737074 |
| rs216497 | G | T | 0.090576 | 0.019773 | 4.63 × 10^−6^ | 0.732557 | 20.98337 | 0.001579 | 0.000937 | 0.092001 | 0.679329 |
| rs2531358 | G | A | −0.08086 | 0.017253 | 2.77 × 10^−6^ | 0.534788 | 21.96748 | −0.00235 | 0.000882 | 0.0077 | 0.443531 |
| rs4739605 | T | C | 0.62815 | 0.13225 | 2.04 × 10^−6^ | 0.993498 | 22.55981 | −0.00058 | 0.0029 | 0.84 | 0.975239 |
| rs58472553 | T | G | 0.16029 | 0.034044 | 2.50 × 10^−6^ | 0.062276 | 22.16852 | −0.00078 | 0.001826 | 0.67 | 0.061026 |
| rs6752506 | G | A | −0.09907 | 0.019331 | 2.97 × 10^−7^ | 0.282251 | 26.26661 | −0.00246 | 0.00101 | 0.015 | 0.253449 |
| rs72994670 | C | T | 0.225847 | 0.046984 | 1.53 × 10^−6^ | 0.032214 | 23.10581 | 0.002632 | 0.002024 | 0.19 | 0.049286 |
| rs76537824 | C | T | 0.278102 | 0.060449 | 4.21 × 10^−6^ | 0.018104 | 21.16592 | 0.003164 | 0.001802 | 0.079001 | 0.066489 |
| rs77392397 | G | C | 0.320963 | 0.069124 | 3.43 × 10^−6^ | 0.013865 | 21.56017 | −0.00121 | 0.002382 | 0.61 | 0.034911 |

EA: Effect allele; OA: Other allele; EAF: Effect allele frequency; TMDs: Temporomandibular disorders; SNP: Single nucleotide polymorphism; SE: Standard Error.

Supplementary Table 4. Detail information of selected SNPs for MR analysis of the causal effect of TMDs on facial pain.

| SNP ID | EA | OA | Exposure | | | | | Outcome | | | |
| --- | --- | --- | --- | --- | --- | --- | --- | --- | --- | --- | --- |
|  |  |  | Beta | SE | *p*-value | EAF | *F* | Beta | SE | *p*-value | EAF |
| rs10882591 | T | C | −0.16265 | 0.031081 | 1.67 × 10^−7^ | 0.090996 | 27.38553 | −0.00016 | 0.000519 | 0.75 | 0.080914 |
| rs112370138 | T | C | −0.22908 | 0.0482 | 2.01 × 10^−6^ | 0.038022 | 22.58832 | −0.00091 | 0.000654 | 0.16 | 0.048757 |
| rs139330 | A | C | 0.084209 | 0.017731 | 2.04 × 10^−6^ | 0.36942 | 22.55519 | −8.34 × 10^−5^ | 0.000322 | 0.8 | 0.258042 |
| rs1960036 | A | C | 0.09776 | 0.021291 | 4.40 × 10^−6^ | 0.198771 | 21.08376 | −0.00013 | 0.000365 | 0.719999 | 0.182171 |
| rs2058908 | C | T | −0.08637 | 0.018036 | 1.68 × 10^−6^ | 0.663399 | 22.93359 | −0.00064 | 0.000319 | 0.047 | 0.737074 |
| rs216497 | G | T | 0.090576 | 0.019773 | 4.63 × 10^−6^ | 0.732557 | 20.98337 | −0.00017 | 0.000301 | 0.58 | 0.679329 |
| rs2531358 | G | A | −0.08086 | 0.017253 | 2.77 × 10^−6^ | 0.534788 | 21.96748 | −0.00017 | 0.000283 | 0.56 | 0.443531 |
| rs58472553 | T | G | 0.16029 | 0.034044 | 2.50 × 10^−6^ | 0.062276 | 22.16852 | 0.000172 | 0.000586 | 0.77 | 0.061026 |
| rs6752506 | G | A | −0.09907 | 0.019331 | 2.97 × 10^−7^ | 0.282251 | 26.26661 | −0.00028 | 0.000324 | 0.39 | 0.253449 |
| rs72994670 | C | T | 0.225847 | 0.046984 | 1.53 × 10^−6^ | 0.032214 | 23.10581 | 0.001247 | 0.00065 | 0.055 | 0.049286 |
| rs76537824 | C | T | 0.278102 | 0.060449 | 4.21 × 10^−6^ | 0.018104 | 21.16592 | 0.000768 | 0.000579 | 0.18 | 0.066489 |

EA: Effect allele; OA: Other allele; EAF: Effect allele frequency; TMDs: Temporomandibular disorders; SNP: Single nucleotide polymorphism; SE: Standard Error.

Supplementary Table 5. Detail information of selected SNPs for MR analysis of the causal effect of TMDs on cluster headache.

| SNP | EA | OA | Exposure | | | | | Outcome | | | |  |
| --- | --- | --- | --- | --- | --- | --- | --- | --- | --- | --- | --- | --- |
|  |  |  | Beta | SE | *p*-value | EAF | *F* | Beta | SE | *p*-value | EAF | |
| rs10882591 | T | C | −0.16265 | 1.67 × 10^−7^ | 0.031081 | 0.090996 | 27.38553 | 0.061062 | 0.067224 | 0.363704 | 0.090914 | |
| rs111626487 | A | G | 0.252379 | 4.18 × 10^−6^ | 0.054841 | 0.022211 | 21.1786 | −0.1014 | 0.129203 | 0.432589 | 0.022338 | |
| rs112370138 | T | C | −0.22908 | 2.01 × 10^−6^ | 0.0482 | 0.038022 | 22.58832 | 0.016503 | 0.100298 | 0.86931 | 0.038091 | |
| rs114568702 | C | T | −0.37685 | 2.45 × 10^−6^ | 0.079973 | 0.015112 | 22.20439 | −0.04467 | 0.160098 | 0.780256 | 0.01483 | |
| rs11675477 | C | A | 0.185278 | 3.32 × 10^−6^ | 0.039844 | 0.94562 | 21.62373 | 0.096457 | 0.085215 | 0.257669 | 0.94578 | |
| rs137964046 | A | G | −0.68804 | 3.04 × 10^−6^ | 0.147387 | 0.005489 | 21.79241 | 0.605262 | 0.229926 | 0.008478 | 0.00534 | |
| rs139330 | A | C | 0.084209 | 2.04 × 10^−6^ | 0.017731 | 0.36942 | 22.55519 | −0.03321 | 0.040103 | 0.407599 | 0.370059 | |
| rs1960036 | A | C | 0.09776 | 4.40 × 10^−6^ | 0.021291 | 0.198771 | 21.08376 | −0.07266 | 0.048561 | 0.13458 | 0.198718 | |
| rs199894590 | T | G | 0.106329 | 1.60 × 10^−6^ | 0.022159 | 0.181694 | 23.02581 | 0.025098 | 0.050954 | 0.62232 | 0.181225 | |
| rs2058908 | C | T | −0.08637 | 1.68 × 10^−6^ | 0.018036 | 0.663399 | 22.93359 | 0.028995 | 0.040914 | 0.478518 | 0.662982 | |
| rs216497 | G | T | 0.090576 | 4.63 × 10^−6^ | 0.019773 | 0.732557 | 20.98337 | 0.015047 | 0.044003 | 0.732384 | 0.733692 | |
| rs2531358 | G | A | −0.08086 | 2.77 × 10^−6^ | 0.017253 | 0.534788 | 21.96748 | −0.09285 | 0.038851 | 0.016858 | 0.534128 | |
| rs4739605 | T | C | 0.62815 | 2.04 × 10^−6^ | 0.13225 | 0.993498 | 22.55981 | 0.177408 | 0.252427 | 0.482174 | 0.993384 | |
| rs58472553 | T | G | 0.16029 | 2.50 × 10^−6^ | 0.034044 | 0.062276 | 22.16852 | −0.08761 | 0.080601 | 0.277081 | 0.062268 | |
| rs6752506 | G | A | −0.09907 | 2.97 × 10^−7^ | 0.019331 | 0.282251 | 26.26661 | −0.04701 | 0.042919 | 0.273395 | 0.282573 | |
| rs72994670 | C | T | 0.225847 | 1.53 × 10^−6^ | 0.046984 | 0.032214 | 23.10581 | −0.15773 | 0.111571 | 0.157457 | 0.031606 | |
| rs76537824 | C | T | 0.278102 | 4.21 × 10^−6^ | 0.060449 | 0.018104 | 21.16592 | 0.10506 | 0.14988 | 0.483326 | 0.01784 | |
| rs77392397 | G | C | 0.320963 | 3.43 × 10^−6^ | 0.069124 | 0.013865 | 21.56017 | −0.10824 | 0.171283 | 0.527446 | 0.013947 | |

EA: Effect allele; OA: Other allele; EAF: Effect allele frequency; TMDs: Temporomandibular disorders; SNP: Single nucleotide polymorphism; SE: Standard Error.

Supplementary Table 6. Detail information of selected SNPs for MR analysis of the causal effect of TMDs on tension-types headache.

| SNP | EA | OA | Exposure | | | | | Outcome | | | |  |
| --- | --- | --- | --- | --- | --- | --- | --- | --- | --- | --- | --- | --- |
|  |  |  | Beta | SE | *p*-value | EAF | *F* | Beta | SE | *p*-value | EAF | |
| rs10882591 | T | C | −0.16265 | 0.031081 | 1.67 × 10^−7^ | 0.090996 | 27.38553 | 0.156 | 0.147 | 0.289 | 0.080938 | |
| rs111626487 | A | G | 0.252379 | 0.054841 | 4.18 × 10^−6^ | 0.022211 | 21.1786 | 0.1 | 0.306 | 0.743 | 0.017528 | |
| rs112370138 | T | C | −0.22908 | 0.0482 | 2.01 × 10^−6^ | 0.038022 | 22.58832 | −0.00983 | 0.184 | 0.957 | 0.049126 | |
| rs114568702 | C | T | −0.37685 | 0.079973 | 2.45 × 10^−6^ | 0.015112 | 22.20439 | 0.337 | 0.29 | 0.244 | 0.020978 | |
| rs11675477 | C | A | 0.185278 | 0.039844 | 3.32 × 10^−6^ | 0.94562 | 21.62373 | −0.129 | 0.211 | 0.542 | 0.962241 | |
| rs137964046 | A | G | −0.68804 | 0.147387 | 3.04 × 10^−6^ | 0.005489 | 21.79241 | −0.15 | 0.443 | 0.736 | 0.01107 | |
| rs139330 | A | C | 0.084209 | 0.017731 | 2.04 × 10^−6^ | 0.36942 | 22.55519 | −0.0119 | 0.0915 | 0.896 | 0.258403 | |
| rs1960036 | A | C | 0.09776 | 0.021291 | 4.40 × 10^−6^ | 0.198771 | 21.08376 | −0.0368 | 0.104 | 0.723 | 0.181665 | |
| rs2058908 | C | T | −0.08637 | 0.018036 | 1.68 × 10^−6^ | 0.663399 | 22.93359 | 0.107 | 0.0904 | 0.235 | 0.734388 | |
| rs216497 | G | T | 0.090576 | 0.019773 | 4.63 × 10^−6^ | 0.732557 | 20.98337 | −0.0567 | 0.0852 | 0.506 | 0.67767 | |
| rs2531358 | G | A | −0.08086 | 0.017253 | 2.77 × 10^−6^ | 0.534788 | 21.96748 | 0.014 | 0.0805 | 0.862 | 0.443371 | |
| rs4739605 | T | C | 0.62815 | 0.13225 | 2.04 × 10^−6^ | 0.993498 | 22.55981 | −0.133 | 0.261 | 0.612 | 0.974618 | |
| rs58472553 | T | G | 0.16029 | 0.034044 | 2.50 × 10^−6^ | 0.062276 | 22.16852 | −0.0112 | 0.167 | 0.947 | 0.060764 | |
| rs6752506 | G | A | −0.09907 | 0.019331 | 2.97 × 10^−6^ | 0.282251 | 26.26661 | 0.0565 | 0.0917 | 0.538 | 0.255079 | |
| rs72994670 | C | T | 0.225847 | 0.046984 | 1.53 × 10^−6^ | 0.032214 | 23.10581 | −0.036 | 0.184 | 0.845 | 0.049435 | |
| rs76537824 | C | T | 0.278102 | 0.060449 | 4.21 × 10^−6^ | 0.018104 | 21.16592 | −0.0534 | 0.164 | 0.745 | 0.066402 | |
| rs77392397 | G | C | 0.320963 | 0.069124 | 3.43 × 10^−6^ | 0.013865 | 21.56017 | −0.368 | 0.218 | 0.0916 | 0.034486 | |

EA: Effect allele; OA: Other allele; EAF: Effect allele frequency; TMDs: Temporomandibular disorders; SNP: Single nucleotide polymorphism; SE: Standard Error.

Supplementary Table 7. Detail information of selected SNPs for MR analysis of the causal effect of TMDs on migraine.

| SNP ID | EA | OA | Exposure | | | | | Outcome | | | |
| --- | --- | --- | --- | --- | --- | --- | --- | --- | --- | --- | --- |
|  |  |  | Beta | SE | *p*-value | EAF | *F* | Beta | SE | *p*-value | EAF |
| rs10882591 | T | C | −0.16265 | 0.031081 | 1.67 × 10^−7^ | 0.090996 | 27.38553 | −0.00018 | 0.000628 | 0.8 | 0.081128 |
| rs111626487 | A | G | 0.252379 | 0.054841 | 4.18 × 10^−6^ | 0.022211 | 21.1786 | 0.002075 | 0.001326 | 0.12 | 0.016796 |
| rs112370138 | T | C | −0.22908 | 0.0482 | 2.01 × 10^−6^ | 0.038022 | 22.58832 | 0.000643 | 0.000803 | 0.43 | 0.047239 |
| rs114568702 | C | T | −0.37685 | 0.079973 | 2.45 × 10^−6^ | 0.015112 | 22.20439 | 0.001238 | 0.001276 | 0.34 | 0.019867 |
| rs11675477 | C | A | 0.185278 | 0.039844 | 3.32 × 10^−6^ | 0.94562 | 21.62373 | −0.00103 | 0.000853 | 0.22 | 0.953507 |
| rs137964046 | A | G | −0.68804 | 0.147387 | 3.04 × 10^−6^ | 0.005489 | 21.79241 | 0.003092 | 0.001912 | 0.1 | 0.010755 |
| rs139330 | A | C | 0.084209 | 0.017731 | 2.04 × 10^−6^ | 0.36942 | 22.55519 | 0.000308 | 0.000392 | 0.43 | 0.255593 |
| rs1960036 | A | C | 0.09776 | 0.021291 | 4.40 × 10^−6^ | 0.198771 | 21.08376 | −0.00113 | 0.000444 | 0.011 | 0.180862 |
| rs199894590 | T | G | 0.106329 | 0.022159 | 1.60 × 10^−6^ | 0.181694 | 23.02581 | −0.00018 | 0.000573 | 0.77 | 0.123604 |
| rs2058908 | C | T | −0.08637 | 0.018036 | 1.68 × 10^−6^ | 0.663399 | 22.93359 | −0.00011 | 0.00039 | 0.780001 | 0.741022 |
| rs216497 | G | T | 0.090576 | 0.019773 | 4.63 × 10^−6^ | 0.732557 | 20.98337 | 0.000175 | 0.000365 | 0.61 | 0.680593 |
| rs2531358 | G | A | −0.08086 | 0.017253 | 2.77 × 10^−6^ | 0.534788 | 21.96748 | 3.55 × 10^−5^ | 0.000343 | 0.91 | 0.442773 |
| rs4739605 | T | C | 0.62815 | 0.13225 | 2.04 × 10^−6^ | 0.993498 | 22.55981 | 0.001205 | 0.001148 | 0.31 | 0.976144 |
| rs58472553 | T | G | 0.16029 | 0.034044 | 2.50 × 10^−6^ | 0.062276 | 22.16852 | −0.00128 | 0.00071 | 0.065999 | 0.060745 |
| rs6752506 | G | A | −0.09907 | 0.019331 | 2.97 × 10^−7^ | 0.282251 | 26.26661 | 7.81 × 10^−5^ | 0.000394 | 0.87 | 0.252163 |
| rs72994670 | C | T | 0.225847 | 0.046984 | 1.53 × 10^−6^ | 0.032214 | 23.10581 | 0.00034 | 0.00077 | 0.66 | 0.05176 |
| rs76537824 | C | T | 0.278102 | 0.060449 | 4.21 × 10^−6^ | 0.018104 | 21.16592 | −0.0005 | 0.000714 | 0.48 | 0.064006 |
| rs77392397 | G | C | 0.320963 | 0.069124 | 3.43 × 10^−6^ | 0.013865 | 21.56017 | 0.000479 | 0.000944 | 0.62 | 0.033522 |

EA: Effect allele; OA: Other allele; EAF: Effect allele frequency; TMDs: Temporomandibular disorders; SNP: Single nucleotide polymorphism; SE: Standard Error.

Supplementary Table 8. Detail information of selected SNPs for MR analysis of the causal effect of headache on TMDs.

| SNP | EA | OA | Exposure | | | | | Outcome | | | |
| --- | --- | --- | --- | --- | --- | --- | --- | --- | --- | --- | --- |
|  |  |  | Beta | SE | *p*-value | EAF | *F* | Beta | SE | *p*-value | EAF |
| rs115573881 | C | T | 0.002161 | 0.0004 | 6.40 × 10^−8^ | 0.060417 | 29.23329 | 0.003703 | 0.0344 | 0.914276 | 0.068397 |
| rs140996669 | A | G | 0.003324 | 0.000667 | 6.20 × 10^−7^ | 0.021819 | 24.83482 | 0.023424 | 0.078235 | 0.764629 | 0.01287 |
| rs73393137 | T | G | −0.00145 | 0.000315 | 4.50 × 10^−6^ | 0.099976 | 21.03644 | −0.02902 | 0.039565 | 0.463252 | 0.051167 |
| rs7543791 | G | T | 0.002226 | 0.000454 | 9.40 × 10^−7^ | 0.048856 | 24.05493 | −0.01125 | 0.028725 | 0.69536 | 0.099784 |
| rs79344266 | T | G | 0.002563 | 0.000525 | 1.10 × 10^−6^ | 0.033197 | 23.80103 | −0.14585 | 0.083324 | 0.080061 | 0.010899 |
| rs9486719 | A | G | 0.001094 | 0.000235 | 3.20 × 10^−6^ | 0.200405 | 21.66523 | −0.00882 | 0.026061 | 0.734981 | 0.125014 |

EA: Effect allele; OA: Other allele; EAF: Effect allele frequency; TMDs: Temporomandibular disorders; SNP: Single nucleotide polymorphism; SE: Standard Error.

Supplementary Table 9. Detail information of selected SNPs for MR analysis of the causal effect of neck/shoulder pain on TMDs.

| SNP | EA | OA | Exposure | | | | | Outcome | | | |
| --- | --- | --- | --- | --- | --- | --- | --- | --- | --- | --- | --- |
|  |  |  | Beta | SE | *p*-value | EAF | *F* | Beta | SE | *p*-value | EAF |
| rs10135643 | C | A | 0.004738 | 0.000907 | 1.80 × 10^−7^ | 0.372296 | 27.27106 | 0.001274 | 0.018195 | 0.944196 | 0.343515 |
| rs1018503 | G | T | −0.00415 | 0.000884 | 2.70 × 10^−6^ | 0.429536 | 22.02984 | 0.011244 | 0.017346 | 0.516836 | 0.446288 |
| rs10789929 | T | C | 0.004763 | 0.000902 | 1.30 × 10^−7^ | 0.38504 | 27.8792 | 0.0493 | 0.018099 | 0.006451 | 0.626682 |
| rs10818936 | G | C | 0.004772 | 0.000942 | 4.10 × 10^−7^ | 0.320277 | 25.66898 | 0.015362 | 0.018414 | 0.404156 | 0.324719 |
| rs11171739 | T | C | 0.004062 | 0.000883 | 4.30 × 10^−6^ | 0.568548 | 21.14205 | 0.025053 | 0.017426 | 0.150516 | 0.56952 |
| rs112039693 | C | T | 0.015601 | 0.003236 | 1.40 × 10^−6^ | 0.019444 | 23.23791 | 0.181387 | 0.091948 | 0.048528 | 0.009335 |
| rs112292112 | A | G | −0.01264 | 0.002552 | 7.30 × 10^−7^ | 0.030415 | 24.52247 | −0.02997 | 0.067919 | 0.659065 | 0.016035 |
| rs11252332 | A | T | −0.00432 | 0.000914 | 2.30 × 10^−6^ | 0.357903 | 22.32759 | −0.00178 | 0.018779 | 0.924381 | 0.302582 |
| rs11736161 | A | G | 0.004329 | 0.000914 | 2.20 × 10^−6^ | 0.640942 | 22.41923 | −0.00779 | 0.018346 | 0.671039 | 0.66737 |
| rs12659211 | A | T | 0.005086 | 0.001071 | 2.00 × 10^−6^ | 0.213274 | 22.56073 | 0.008069 | 0.019012 | 0.671282 | 0.294052 |
| rs12732083 | T | C | 0.00524 | 0.001047 | 5.60 × 10^−7^ | 0.226532 | 25.06166 | 0.000218 | 0.020522 | 0.991512 | 0.231046 |
| rs1286231 | C | T | −0.00423 | 0.000896 | 2.30 × 10^−6^ | 0.588408 | 22.2936 | 0.07477 | 0.017528 | 1.99 × 10^−5^ | 0.583082 |
| rs13031851 | C | T | −0.00459 | 0.0009 | 3.50 × 10^−7^ | 0.380864 | 25.93832 | −0.03457 | 0.018045 | 0.055386 | 0.353182 |
| rs13107325 | T | C | 0.010907 | 0.001665 | 5.70 × 10^−11^ | 0.074888 | 42.93217 | 0.190335 | 0.07513 | 0.011296 | 0.014052 |
| rs1338034 | A | G | 0.004238 | 0.000899 | 2.50 × 10^−6^ | 0.602822 | 22.2038 | 0.008073 | 0.01786 | 0.651253 | 0.627515 |
| rs1399629 | A | G | −0.00445 | 0.000874 | 3.70 × 10^−7^ | 0.507288 | 25.84172 | −0.02936 | 0.017245 | 0.08865 | 0.480622 |
| rs1476535 | T | C | 0.00489 | 0.000888 | 3.70 × 10^−8^ | 0.561666 | 30.32335 | 0.040727 | 0.017288 | 0.018484 | 0.521969 |
| rs1532204 | G | C | −0.00587 | 0.001193 | 8.70 × 10^−7^ | 0.839533 | 24.1971 | −0.07892 | 0.027874 | 0.004634 | 0.894318 |
| rs159963 | A | C | −0.00437 | 0.000891 | 9.50 × 10^−7^ | 0.581595 | 24.02026 | −0.05552 | 0.018145 | 0.002213 | 0.657013 |
| rs16980973 | T | A | −0.00693 | 0.00132 | 1.50 × 10^−7^ | 0.126644 | 27.53774 | −0.00649 | 0.023021 | 0.777897 | 0.169063 |
| rs1962104 | C | T | 0.004122 | 0.000889 | 3.50 × 10^−6^ | 0.558211 | 21.49408 | 0.018166 | 0.017284 | 0.293257 | 0.475843 |
| rs1963206 | A | G | −0.00538 | 0.001064 | 4.30 × 10^−7^ | 0.782775 | 25.55661 | −0.03629 | 0.019843 | 0.067386 | 0.748464 |
| rs198230 | C | A | −0.005 | 0.001054 | 2.10 × 10^−6^ | 0.777437 | 22.52346 | −0.00806 | 0.018466 | 0.662421 | 0.677629 |
| rs2149814 | C | A | 0.006626 | 0.001362 | 1.10 × 10^−6^ | 0.118568 | 23.67038 | −0.01211 | 0.029787 | 0.684443 | 0.091746 |
| rs2283076 | G | A | 0.005173 | 0.001045 | 7.50 × 10^−7^ | 0.22942 | 24.49265 | 0.02235 | 0.024089 | 0.353509 | 0.152912 |
| rs2587363 | A | G | −0.00443 | 0.000885 | 5.40 × 10^−7^ | 0.569798 | 25.12913 | −0.01842 | 0.01737 | 0.288889 | 0.56872 |
| rs270833 | G | A | −0.00412 | 0.000877 | 2.60 × 10^−6^ | 0.47098 | 22.09104 | −0.02175 | 0.017292 | 0.208469 | 0.537295 |
| rs274622 | T | C | −0.00426 | 0.00092 | 3.70 × 10^−6^ | 0.652806 | 21.40897 | −0.00472 | 0.017671 | 0.789416 | 0.605431 |
| rs2898518 | A | G | 0.004078 | 0.000891 | 4.70 × 10^−6^ | 0.413944 | 20.96293 | 0.030438 | 0.017775 | 0.086828 | 0.37847 |
| rs2919935 | T | A | 0.007877 | 0.001642 | 1.60 × 10^−6^ | 0.077051 | 23.0207 | −0.05733 | 0.029164 | 0.049305 | 0.096516 |
| rs3132469 | G | A | 0.005705 | 0.001227 | 3.30 × 10^−6^ | 0.850998 | 21.63405 | 0.016796 | 0.025339 | 0.507424 | 0.86819 |
| rs34332831 | T | C | 0.005923 | 0.001283 | 3.90 × 10^−6^ | 0.134811 | 21.32493 | 0.052531 | 0.025777 | 0.04156 | 0.12844 |
| rs35919525 | G | T | 0.008331 | 0.001775 | 2.70 × 10^−6^ | 0.066366 | 22.04156 | 0.008583 | 0.030621 | 0.779258 | 0.086655 |
| rs36042757 | C | A | −0.00687 | 0.001274 | 7.00 × 10^−8^ | 0.137385 | 29.05402 | −0.0381 | 0.029876 | 0.20218 | 0.092027 |
| rs4141466 | C | T | 0.004447 | 0.000937 | 2.10 × 10^−6^ | 0.327824 | 22.54145 | 0.003888 | 0.017843 | 0.827513 | 0.368179 |
| rs4518923 | G | A | −0.00473 | 0.000929 | 3.50 × 10^−7^ | 0.350504 | 25.93378 | 0.017179 | 0.017597 | 0.328921 | 0.413437 |
| rs4886860 | C | G | −0.00548 | 0.001035 | 1.20 × 10^−7^ | 0.767425 | 28.00519 | −0.00415 | 0.018816 | 0.825617 | 0.698904 |
| rs55670730 | T | A | 0.0066 | 0.00141 | 2.90 × 10^−6^ | 0.110749 | 21.91179 | 0.021258 | 0.027675 | 0.442421 | 0.10949 |
| rs55886108 | T | C | −0.00415 | 0.0009 | 3.90 × 10^−6^ | 0.388443 | 21.31086 | −0.0116 | 0.018201 | 0.523892 | 0.339777 |
| rs56069701 | T | C | −0.00726 | 0.001542 | 2.50 × 10^−6^ | 0.093227 | 22.16774 | 0.065285 | 0.046396 | 0.159395 | 0.036522 |
| rs568809 | G | C | 0.00549 | 0.001119 | 9.20 × 10^−7^ | 0.808201 | 24.08356 | −0.00762 | 0.02647 | 0.773462 | 0.878523 |
| rs58621819 | T | A | 0.005192 | 0.001064 | 1.10 × 10^−6^ | 0.217556 | 23.83324 | 0.027393 | 0.030654 | 0.37152 | 0.089712 |
| rs6041749 | C | T | 0.004556 | 0.000932 | 1.00 × 10^−6^ | 0.440756 | 23.91464 | 0.001584 | 0.018886 | 0.933148 | 0.32373 |
| rs6062964 | T | C | −0.02085 | 0.00409 | 3.50 × 10^−7^ | 0.011778 | 25.97624 | 0.106723 | 0.088238 | 0.226473 | 0.00957 |
| rs6119944 | G | A | 0.005294 | 0.001041 | 3.60 × 10^−7^ | 0.239843 | 25.88159 | 0.001416 | 0.018657 | 0.9395 | 0.313377 |
| rs61779314 | T | C | 0.006177 | 0.001128 | 4.30 × 10^−8^ | 0.185979 | 30.00269 | −0.00431 | 0.021512 | 0.841311 | 0.20213 |
| rs62056490 | A | C | −0.0062 | 0.001089 | 1.20 × 10^−8^ | 0.794202 | 32.45511 | −0.018 | 0.023869 | 0.450697 | 0.843066 |
| rs62131354 | T | C | 0.004821 | 0.001048 | 4.20 × 10^−6^ | 0.2257 | 21.16933 | 0.034125 | 0.01881 | 0.069642 | 0.302659 |
| rs6606710 | C | T | 0.004566 | 0.000922 | 7.40 × 10^−7^ | 0.406931 | 24.51218 | −0.00138 | 0.018545 | 0.940678 | 0.332556 |
| rs6935368 | T | C | −0.00454 | 0.000904 | 5.10 × 10^−7^ | 0.380828 | 25.23667 | 0.028319 | 0.017486 | 0.10534 | 0.429564 |
| rs71607173 | G | C | 0.016426 | 0.003533 | 3.30 × 10^−6^ | 0.016225 | 21.61591 | 0.052262 | 0.0719 | 0.467309 | 0.014733 |
| rs7232237 | G | A | 0.004682 | 0.000875 | 8.80 × 10^−8^ | 0.501939 | 28.62137 | −0.02065 | 0.017292 | 0.23243 | 0.538187 |
| rs7248205 | T | C | −0.00502 | 0.000896 | 2.10 × 10^−8^ | 0.600262 | 31.42091 | 0.008667 | 0.017675 | 0.623883 | 0.607826 |
| rs72671456 | A | G | 0.006586 | 0.001282 | 2.80 × 10^−7^ | 0.135444 | 26.39565 | 0.010709 | 0.029413 | 0.715784 | 0.095785 |
| rs729053 | C | G | 0.005082 | 0.000932 | 5.00 × 10^−8^ | 0.332118 | 29.71121 | −0.02363 | 0.01863 | 0.204691 | 0.31265 |
| rs7297794 | T | C | −0.00512 | 0.001052 | 1.10 × 10^−6^ | 0.224021 | 23.66614 | −0.06422 | 0.019719 | 0.001128 | 0.265847 |
| rs7317962 | T | G | 0.004724 | 0.000895 | 1.30 × 10^−7^ | 0.605114 | 27.85487 | −0.01079 | 0.017341 | 0.533769 | 0.530716 |
| rs7739896 | G | A | −0.00418 | 0.000878 | 1.90 × 10^−6^ | 0.531089 | 22.6909 | 0.003833 | 0.017231 | 0.823977 | 0.495721 |
| rs78169991 | G | A | −0.01454 | 0.002722 | 9.10 × 10^−8^ | 0.027472 | 28.55927 | −0.04767 | 0.050361 | 0.343904 | 0.030586 |
| rs8054093 | C | A | −0.00431 | 0.000892 | 1.30 × 10^−6^ | 0.563325 | 23.41735 | −0.02778 | 0.018068 | 0.124104 | 0.643689 |
| rs9375610 | G | A | 0.004078 | 0.000888 | 4.40 × 10^−6^ | 0.580922 | 21.07859 | −0.00657 | 0.017778 | 0.711684 | 0.619222 |
| rs9889282 | C | A | 0.006232 | 0.000902 | 4.80 × 10^−12^ | 0.387918 | 47.75088 | 0.024004 | 0.017882 | 0.179493 | 0.370629 |

EA: Effect allele; OA: Other allele; EAF: Effect allele frequency; TMDs: Temporomandibular disorders; SNP: Single nucleotide polymorphism; SE: Standard Error.

Supplementary Table 10. Detail information of selected SNPs for MR analysis of the causal effect of facial pain on TMDs.

| SNP | EA | OA | Exposure | | | | | Outcome | | | |
| --- | --- | --- | --- | --- | --- | --- | --- | --- | --- | --- | --- |
|  |  |  | Beta | SE | *p*-value | EAF | *F* | Beta | SE | *p*-value | EAF |
| rs12445269 | T | C | −0.00147 | 0.000286 | 2.90 × 10^−7^ | 0.41534 | 26.33085 | 0.00084 | 0.017878 | 0.962503 | 0.371326 |
| rs12712737 | G | A | 0.001344 | 0.000287 | 2.70 × 10^−6^ | 0.420497 | 22.00116 | 0.002434 | 0.017598 | 0.889986 | 0.401037 |
| rs2199214 | C | T | 0.001719 | 0.000374 | 4.40 × 10^−6^ | 0.172202 | 21.07934 | −0.00056 | 0.023495 | 0.980983 | 0.162885 |
| rs34234617 | G | C | −0.00305 | 0.000648 | 2.50 × 10^−6^ | 0.051931 | 22.20504 | 0.039825 | 0.034805 | 0.252527 | 0.066176 |
| rs34311004 | C | T | 0.003352 | 0.000711 | 2.40 × 10^−6^ | 0.042334 | 22.24341 | −0.00179 | 0.054733 | 0.97393 | 0.025642 |
| rs4416176 | C | T | 0.002218 | 0.000477 | 3.30 × 10^−6^ | 0.095787 | 21.62604 | −0.02631 | 0.033282 | 0.429152 | 0.07378 |
| rs6967391 | T | A | 0.001753 | 0.00036 | 1.10 × 10^−6^ | 0.195562 | 23.67744 | −0.01257 | 0.019922 | 0.528052 | 0.256822 |
| rs72694537 | T | C | 0.00164 | 0.000355 | 3.80 × 10^−6^ | 0.199463 | 21.34035 | −0.02956 | 0.02273 | 0.193407 | 0.174895 |
| rs72711207 | C | T | −0.00228 | 0.000476 | 1.70 × 10^−6^ | 0.0976 | 22.95158 | −0.06492 | 0.033724 | 0.05423 | 0.072255 |
| rs76714322 | T | C | 0.002189 | 0.000458 | 1.70 × 10^−6^ | 0.105959 | 22.87755 | −0.01488 | 0.025271 | 0.556036 | 0.134988 |
| rs7937459 | A | C | 0.001399 | 0.000291 | 1.60 × 10^−6^ | 0.402997 | 23.05739 | 0.008686 | 0.017383 | 0.617312 | 0.458048 |
| rs7965652 | A | G | 0.001379 | 0.000293 | 2.60 × 10^−6^ | 0.354152 | 22.09799 | 0.011907 | 0.019273 | 0.536691 | 0.278862 |
| rs8003983 | A | C | −0.00193 | 0.000407 | 2.20 × 10^−6^ | 0.1504 | 22.3747 | 0.03003 | 0.025026 | 0.230155 | 0.141177 |
| rs8005612 | C | T | −0.00166 | 0.000349 | 1.90 × 10^−6^ | 0.207409 | 22.69808 | 0.002739 | 0.021504 | 0.898633 | 0.200978 |
| rs9643685 | T | C | −0.00153 | 0.000333 | 4.30 × 10^−6^ | 0.765689 | 21.12775 | −0.01385 | 0.023511 | 0.555834 | 0.840191 |
| rs9645327 | T | C | 0.002054 | 0.00044 | 3.00 × 10^−6^ | 0.117246 | 21.82874 | 0.003028 | 0.025564 | 0.905706 | 0.129289 |

EA: Effect allele; OA: Other allele; EAF: Effect allele frequency; TMDs: Temporomandibular disorders; SNP: Single nucleotide polymorphism; SE: Standard Error.

Supplementary Table 11. Detail information of selected SNPs for MR analysis of the causal effect of cluster headache on TMDs.

| SNP | EA | OA | Exposure | | | | | Outcome | | | |
| --- | --- | --- | --- | --- | --- | --- | --- | --- | --- | --- | --- |
|  |  |  | Beta | SE | *p*-value | EAF | *F* | Beta | SE | *p*-value | EAF |
| rs111389066 | C | G | −1.12758 | 0.242603 | 3.35 × 10^−6^ | 0.012873 | 21.60242 | 0.091666 | 0.077217 | 0.235185 | 0.012979 |
| rs118067737 | T | A | −1.33881 | 0.28874 | 3.54 × 10^−6^ | 0.01003 | 21.49928 | 0.086704 | 0.088153 | 0.325329 | 0.009917 |
| rs118091971 | A | G | 0.520692 | 0.112553 | 3.72 × 10^−6^ | 0.022599 | 21.40167 | 0.000194 | 0.058305 | 0.997345 | 0.022544 |
| rs141034388 | T | C | 0.523304 | 0.108484 | 1.41 × 10^−6^ | 0.023735 | 23.26895 | 0.028908 | 0.057413 | 0.614611 | 0.023934 |
| rs185426866 | T | A | 0.593014 | 0.121285 | 1.01 × 10^−6^ | 0.018514 | 23.90648 | 0.036838 | 0.064846 | 0.569982 | 0.018544 |
| rs200795716 | G | A | 0.448835 | 0.092166 | 1.12 × 10^−6^ | 0.033698 | 23.71564 | −0.03781 | 0.048363 | 0.434297 | 0.033538 |
| rs372466710 | C | T | −0.68963 | 0.141341 | 1.07 × 10^−6^ | 0.039839 | 23.80659 | 0.021863 | 0.053711 | 0.683973 | 0.039907 |
| rs3845509 | C | A | 0.192701 | 0.04175 | 3.92 × 10^−6^ | 0.270675 | 21.30349 | −0.02002 | 0.019359 | 0.301095 | 0.27049 |
| rs4560253 | G | C | −0.21466 | 0.042233 | 3.72 × 10^−7^ | 0.318889 | 25.83428 | −0.01074 | 0.018523 | 0.562062 | 0.318671 |
| rs6135407 | T | C | −0.26107 | 0.052567 | 6.82 × 10^−7^ | 0.178351 | 24.66489 | −0.01206 | 0.022587 | 0.593402 | 0.178425 |
| rs6508354 | C | T | 0.243459 | 0.050869 | 1.70 × 10^−6^ | 0.147766 | 22.90588 | 0.011647 | 0.024253 | 0.631066 | 0.148161 |
| rs7291345 | C | G | −0.28026 | 0.060235 | 3.28 × 10^−6^ | 0.13215 | 21.64787 | 0.020477 | 0.025397 | 0.420068 | 0.13219 |
| rs73083559 | T | C | −2.89007 | 0.605983 | 1.85 × 10^−6^ | 0.005394 | 22.74552 | −0.02148 | 0.118167 | 0.855758 | 0.005559 |
| rs73310223 | T | A | 0.579721 | 0.124217 | 3.06 × 10^−6^ | 0.017241 | 21.78091 | 0.207107 | 0.06243 | 0.000908 | 0.017396 |
| rs78350310 | T | C | 0.392327 | 0.08542 | 4.37 × 10^−6^ | 0.043127 | 21.09474 | 0.039372 | 0.042678 | 0.356249 | 0.042971 |

EA: Effect allele; OA: Other allele; EAF: Effect allele frequency; TMDs: Temporomandibular disorders; SNP: Single nucleotide polymorphism; SE: Standard Error.

Supplementary Table 12. Detail information of selected SNPs for MR analysis of the causal effect of tension-types headache on TMDs.

| SNP | EA | OA | Exposure | | | | | Outcome | | | |
| --- | --- | --- | --- | --- | --- | --- | --- | --- | --- | --- | --- |
|  |  |  | Beta | SE | *p*-value | EAF | *F* | Beta | SE | *p*-value | EAF |
| rs12349132 | T | C | 2.13 | 0.466 | 4.80 × 10^−6^ | 0.010762 | 20.89235 | 0.291502 | 0.191451 | 0.127859 | 0.002253 |
| rs139386387 | A | G | 1.32 | 0.287 | 4.38 × 10^−6^ | 0.02713 | 21.15359 | −0.00484 | 0.080822 | 0.95224 | 0.011931 |
| rs140562886 | T | C | 1.85 | 0.404 | 4.74 × 10^−6^ | 0.014439 | 20.96915 | 0.273077 | 0.172537 | 0.113487 | 0.002538 |
| rs28699426 | A | T | 0.415 | 0.09 | 3.99 × 10^−6^ | 0.282978 | 21.26235 | 0.008493 | 0.020236 | 0.674704 | 0.24578 |
| rs3810037 | T | C | 0.45 | 0.091 | 7.59 × 10^−7^ | 0.278832 | 24.45357 | 0.008339 | 0.018968 | 0.660192 | 0.294455 |
| rs4784924 | T | G | −0.401 | 0.0833 | 1.48 × 10^−6^ | 0.625173 | 23.17388 | −0.00451 | 0.017783 | 0.79993 | 0.610578 |
| rs58692695 | A | G | 0.662 | 0.133 | 7.14 × 10^−7^ | 0.115647 | 24.77494 | 0.04217 | 0.036104 | 0.242802 | 0.062334 |
| rs61992090 | C | T | 1.63 | 0.335 | 1.06 × 10^−6^ | 0.019763 | 23.67476 | 0.140055 | 0.119613 | 0.241638 | 0.005293 |

EA: Effect allele; OA: Other allele; EAF: Effect allele frequency; TMDs: Temporomandibular disorders; SNP: Single nucleotide polymorphism; SE: Standard Error.

Supplementary Table 13. Detail information of selected SNPs for MR analysis of the causal effect of migraine on TMDs.

| SNP | EA | OA | Exposure | | | | | Outcome | | | |  |
| --- | --- | --- | --- | --- | --- | --- | --- | --- | --- | --- | --- | --- |
|  |  |  | Beta | SE | *p*-value | EAF | *F* | Beta | SE | *p*-value | EAF | |
| rs10023050 | G | A | −0.00167 | 0.000349 | 1.40 × 10^−6^ | 0.39164 | 22.84381 | 0.087006 | 0.058692 | 0.13823 | 0.467942 | |
| rs10127740 | T | G | −0.00169 | 0.000371 | 4.40 × 10^−6^ | 0.310689 | 20.7358 | −0.00362 | 0.065079 | 0.955587 | 0.280027 | |
| rs10218452 | G | A | 0.004788 | 0.000407 | 7.40 × 10^−32^ | 0.229834 | 138.5081 | 0.011413 | 0.067945 | 0.866602 | 0.245184 | |
| rs1047891 | A | C | 0.0017 | 0.000365 | 3.20 × 10^−6^ | 0.315455 | 21.6511 | −0.04311 | 0.062385 | 0.489587 | 0.32225 | |
| rs10927722 | A | G | 0.001814 | 0.000356 | 3.20 × 10^−7^ | 0.365356 | 25.99966 | −0.05033 | 0.064951 | 0.438439 | 0.282408 | |
| rs11153082 | G | A | 0.003417 | 0.000364 | 5.20 × 10^−21^ | 0.325011 | 88.30689 | −0.06307 | 0.063616 | 0.32151 | 0.298545 | |
| rs11172113 | C | T | −0.00392 | 0.000346 | 7.60 × 10^−30^ | 0.411057 | 128.6477 | 0.016873 | 0.05958 | 0.777025 | 0.399436 | |
| rs11602707 | T | C | 0.001906 | 0.000401 | 2.10 × 10^−6^ | 0.238508 | 22.56948 | 0.128564 | 0.066644 | 0.053717 | 0.258574 | |
| rs11853918 | T | C | 0.001781 | 0.000381 | 2.70 × 10^−6^ | 0.278792 | 21.83689 | −0.08446 | 0.065352 | 0.196236 | 0.277785 | |
| rs12070846 | C | T | 0.00208 | 0.000409 | 3.40 × 10^−7^ | 0.227741 | 25.83472 | 0.100535 | 0.075594 | 0.183541 | 0.186875 | |
| rs12134493 | A | C | 0.004167 | 0.000533 | 7.10 × 10^−15^ | 0.116843 | 61.14325 | −0.01101 | 0.085216 | 0.897212 | 0.137106 | |
| rs12295710 | T | C | 0.002062 | 0.000342 | 2.00 × 10^−9^ | 0.46249 | 36.26075 | −0.03009 | 0.058364 | 0.60615 | 0.47823 | |
| rs12452590 | G | T | 0.001731 | 0.000361 | 1.30 × 10^−6^ | 0.357283 | 23.04152 | 0.08485 | 0.059208 | 0.151835 | 0.425037 | |
| rs12684144 | C | T | 0.003524 | 0.000406 | 4.30 × 10^−18^ | 0.227875 | 75.43799 | 0.021 | 0.071287 | 0.768318 | 0.213686 | |
| rs12789511 | A | G | 0.001943 | 0.000382 | 3.30 × 10^−7^ | 0.305081 | 25.91045 | 0.146564 | 0.065003 | 0.02415 | 0.277694 | |
| rs12882067 | A | C | −0.00165 | 0.000358 | 4.80 × 10^−6^ | 0.644006 | 21.19932 | 0.12196 | 0.058674 | 0.037655 | 0.543832 | |
| rs12898361 | T | C | 0.001682 | 0.000346 | 1.10 × 10^−6^ | 0.552977 | 23.68774 | 0.113787 | 0.059575 | 0.056135 | 0.583381 | |
| rs12903810 | T | C | 0.002077 | 0.000401 | 3.00 × 10^−7^ | 0.235965 | 26.75729 | −0.01858 | 0.063957 | 0.771418 | 0.292154 | |
| rs13078967 | C | A | −0.00569 | 0.001139 | 7.10 × 10^−7^ | 0.023504 | 24.94672 | −0.42517 | 0.143555 | 0.003059 | 0.051935 | |
| rs149844910 | T | A | 0.004574 | 0.000982 | 3.30 × 10^−6^ | 0.032232 | 21.69751 | 0.049354 | 0.134925 | 0.714523 | 0.048412 | |
| rs16914944 | T | C | −0.00213 | 0.000394 | 5.70 × 10^−8^ | 0.250015 | 29.27176 | −0.02984 | 0.0645 | 0.643664 | 0.28821 | |
| rs17298647 | C | G | 0.002729 | 0.000531 | 2.50 × 10^−7^ | 0.116055 | 26.4234 | −0.04217 | 0.106658 | 0.692575 | 0.081209 | |
| rs181141583 | C | T | −0.0021 | 0.000438 | 1.40 × 10^−6^ | 0.192938 | 22.9744 | 0.143719 | 0.084864 | 0.090353 | 0.13877 | |
| rs2072151 | C | T | 0.00233 | 0.000509 | 4.40 × 10^−6^ | 0.133732 | 20.9511 | 0.004437 | 0.084274 | 0.958016 | 0.138588 | |
| rs2080685 | G | T | 0.002278 | 0.000347 | 5.40 × 10^−11^ | 0.476456 | 43.13956 | −0.03555 | 0.058573 | 0.543865 | 0.51111 | |
| rs227890 | A | G | 0.001819 | 0.000363 | 5.20 × 10^−7^ | 0.340482 | 25.07991 | 0.012432 | 0.06476 | 0.84777 | 0.287354 | |
| rs2294898 | T | G | −0.00271 | 0.000536 | 5.10 × 10^−7^ | 0.113563 | 25.57964 | −0.02317 | 0.080514 | 0.773562 | 0.15346 | |
| rs2317130 | T | C | 0.00175 | 0.000372 | 2.80 × 10^−6^ | 0.70118 | 22.14461 | −0.06081 | 0.064941 | 0.349066 | 0.720476 | |
| rs2840979 | A | G | 0.0017 | 0.000346 | 8.40 × 10^−7^ | 0.434618 | 24.18001 | −0.03904 | 0.05848 | 0.504386 | 0.483952 | |
| rs28451064 | A | G | −0.00279 | 0.000523 | 7.40 × 10^−8^ | 0.126988 | 28.54511 | 0.041509 | 0.081135 | 0.608927 | 0.153385 | |
| rs28731773 | C | T | 0.00241 | 0.000514 | 2.90 × 10^−6^ | 0.12801 | 21.97436 | 0.210291 | 0.119809 | 0.079221 | 0.065445 | |
| rs28929474 | T | C | 0.006168 | 0.001249 | 8.00 × 10^−7^ | 0.01893 | 24.39508 | −0.09799 | 0.210054 | 0.640873 | 0.019777 | |
| rs2905065 | T | C | −0.00185 | 0.000362 | 4.20 × 10^−7^ | 0.668718 | 26.033 | −0.04379 | 0.060664 | 0.470417 | 0.625982 | |
| rs34472962 | T | C | 0.004864 | 0.001061 | 4.00 × 10^−6^ | 0.029948 | 21.00345 | 0.108437 | 0.171212 | 0.526506 | 0.03073 | |
| rs35266980 | T | C | 0.002017 | 0.000371 | 5.10 × 10^−8^ | 0.310822 | 29.60544 | −0.02014 | 0.060049 | 0.737283 | 0.381648 | |
| rs4145901 | G | A | −0.00212 | 0.000406 | 1.90 × 10^−7^ | 0.769732 | 27.40681 | 0.005791 | 0.070567 | 0.934599 | 0.783096 | |
| rs4668251 | G | C | 0.001868 | 0.000376 | 5.30 × 10^−7^ | 0.694853 | 24.74388 | 0.029572 | 0.06774 | 0.662435 | 0.753687 | |
| rs4940804 | T | C | −0.0022 | 0.00042 | 1.40 × 10^−7^ | 0.206949 | 27.4537 | −0.02 | 0.067907 | 0.768401 | 0.244994 | |
| rs57943154 | A | C | 0.005364 | 0.001111 | 1.50 × 10^−6^ | 0.026322 | 23.32176 | 0.214008 | 0.228803 | 0.349613 | 0.016386 | |
| rs6088409 | G | A | 0.001846 | 0.000381 | 1.20 × 10^−6^ | 0.288645 | 23.45579 | 0.079429 | 0.064656 | 0.219263 | 0.286175 | |
| rs61747468 | A | G | 0.00613 | 0.001303 | 3.10 × 10^−6^ | 0.018096 | 22.13349 | −0.19094 | 0.200138 | 0.340061 | 0.021941 | |
| rs6432057 | A | G | −0.00174 | 0.000362 | 1.70 × 10^−6^ | 0.668861 | 23.04958 | −0.0085 | 0.063237 | 0.893105 | 0.698721 | |
| rs6700679 | T | C | −0.00251 | 0.000359 | 2.10 × 10^−12^ | 0.647038 | 49.15961 | 0.031876 | 0.059082 | 0.58953 | 0.579712 | |
| rs6738979 | G | A | −0.0033 | 0.000428 | 1.40 × 10^−14^ | 0.214035 | 59.73908 | 0.053921 | 0.080227 | 0.501519 | 0.157556 | |
| rs6815864 | C | T | 0.002223 | 0.000415 | 7.70 × 10^−8^ | 0.218767 | 28.69334 | 0.135278 | 0.074046 | 0.067706 | 0.191229 | |
| rs71558798 | G | A | 0.003437 | 0.000688 | 5.60 × 10^−7^ | 0.067766 | 24.96708 | 0.07562 | 0.134062 | 0.572708 | 0.051158 | |
| rs73138150 | T | A | 0.00188 | 0.000369 | 3.30 × 10^−7^ | 0.314968 | 25.9194 | 0.050289 | 0.061134 | 0.410735 | 0.355518 | |
| rs73196261 | G | C | −0.00774 | 0.001586 | 9.10 × 10^−7^ | 0.012061 | 23.78001 | −0.50307 | 0.579833 | 0.385604 | 0.002652 | |
| rs7757975 | T | G | 0.003328 | 0.000464 | 1.10 × 10^−12^ | 0.160036 | 51.398 | 0.169296 | 0.093848 | 0.071241 | 0.11074 | |
| rs7758604 | C | T | 0.001963 | 0.000427 | 4.00 × 10^−6^ | 0.203395 | 21.17618 | −0.0787 | 0.068935 | 0.253585 | 0.235088 | |
| rs79486579 | C | A | 0.004368 | 0.000552 | 3.40 × 10^−15^ | 0.107432 | 62.5131 | 0.076769 | 0.081869 | 0.348398 | 0.149084 | |
| rs8075138 | T | C | 0.001613 | 0.000352 | 4.60 × 10^−6^ | 0.392269 | 20.95387 | 0.023254 | 0.059889 | 0.697806 | 0.391707 | |
| rs827396 | T | C | −0.0018 | 0.000376 | 1.90 × 10^−6^ | 0.707575 | 22.83055 | −0.00254 | 0.065454 | 0.969077 | 0.726909 | |
| rs903808 | C | T | −0.00208 | 0.000356 | 5.70 × 10^−9^ | 0.635526 | 33.99467 | 0.004232 | 0.061495 | 0.945136 | 0.648725 | |
| rs914738 | C | T | −0.00184 | 0.000405 | 4.70 × 10^−6^ | 0.69359 | 20.69426 | −0.05285 | 0.061998 | 0.393941 | 0.639346 | |
| rs9349379 | G | A | −0.00319 | 0.000349 | 5.60 × 10^20^ | 0.402367 | 83.57501 | −0.15458 | 0.05783 | 0.007518 | 0.452261 | |
| rs9847964 | T | C | 0.001811 | 0.000369 | 1.00 × 10^−6^ | 0.323769 | 24.04911 | −0.02762 | 0.061228 | 0.651885 | 0.353079 | |

EA: Effect allele; OA: Other allele; EAF: Effect allele frequency; TMDs: Temporomandibular disorders; SNP: Single nucleotide polymorphism; SE: Standard Error.

Supplementary Table 14. Detail information of selected SNPs for MR analysis of the causal effect of bruxism on headache.

| SNP | EA | OA | Exposure | | | | | Outcome | | | |
| --- | --- | --- | --- | --- | --- | --- | --- | --- | --- | --- | --- |
|  |  |  | Beta | SE | *p*-value | EAF | *F* | Beta | SE | *p*-value | EAF |
| rs117019503 | T | A | 0.323912 | 0.068172 | 2.02 × 10^−6^ | 0.195205 | 22.57601 | −7.16 × 10^−5^ | 0.000511 | 0.89 | 0.04922 |
| rs139685925 | A | G | 0.501149 | 0.105944 | 2.24 × 10^−6^ | 0.057895 | 22.37593 | −7.80 × 10^−5^ | 0.000696 | 0.91 | 0.020505 |
| rs1750430 | C | T | 0.436331 | 0.090354 | 1.37 × 10^−6^ | 0.085729 | 23.32042 | −0.00049 | 0.000292 | 0.096 | 0.126366 |
| rs5765490 | C | T | −0.28217 | 0.056989 | 7.37 × 10^−7^ | 0.586109 | 24.51509 | −7.61 × 10^−5^ | 0.000193 | 0.69 | 0.613736 |
| rs76371754 | T | C | −0.77514 | 0.157284 | 8.30 × 10^−7^ | 0.05732 | 24.28797 | −8.49 × 10^−5^ | 0.000461 | 0.85 | 0.050205 |
| rs76578522 | G | A | −1.97938 | 0.417324 | 2.11 × 10^−6^ | 0.015821 | 22.49633 | −0.00035 | 0.000418 | 0.4 | 0.053765 |
| rs78160994 | G | A | 1.89455 | 0.405965 | 3.06 × 10^−6^ | 0.001349 | 21.77885 | 0.000825 | 0.000913 | 0.37 | 0.011044 |
| rs78709176 | C | T | −0.78321 | 0.165384 | 2.18 × 10^−6^ | 0.04841 | 22.4268 | 0.000567 | 0.000534 | 0.29 | 0.031969 |
| rs9597498 | A | G | −0.28285 | 0.061806 | 4.73 × 10^−6^ | 0.339862 | 20.9435 | −0.00014 | 0.0002 | 0.47 | 0.339024 |

EA: Effect allele; OA: Other allele; EAF: Effect allele frequency; SNP: Single nucleotide polymorphism; SE: Standard Error.

Supplementary Table 15. Detail information of selected SNPs for MR analysis of the causal effect of bruxism on neck/shoulder pain.

| SNP | EA | OA | Exposure | | | | | Outcome | | | |
| --- | --- | --- | --- | --- | --- | --- | --- | --- | --- | --- | --- |
|  |  |  | Beta | SE | *p*-value | EAF | *F* | Beta | SE | *p*-value | EAF |
| rs117019503 | T | A | 0.323912 | 0.068172 | 2.02 × 10^−6^ | 0.195205 | 22.57601 | 0.000556 | 0.001343 | 0.68 | 0.122385 |
| rs139685925 | A | G | 0.501149 | 0.105944 | 2.24 × 10^−6^ | 0.057895 | 22.37593 | −0.0026 | 0.003236 | 0.42 | 0.020583 |
| rs144481227 | A | G | 0.836264 | 0.179232 | 3.07 × 10^−6^ | 0.014637 | 21.76986 | 0.007151 | 0.005613 | 0.2 | 0.007393 |
| rs1750430 | C | T | 0.436331 | 0.090354 | 1.37 × 10^−6^ | 0.085729 | 23.32042 | 0.00305 | 0.001384 | 0.028 | 0.116128 |
| rs5765490 | C | T | −0.28217 | 0.056989 | 7.37 × 10^−7^ | 0.586109 | 24.51509 | −0.00029 | 0.000898 | 0.74 | 0.607931 |
| rs76371754 | T | C | −0.77514 | 0.157284 | 8.30 × 10^−7^ | 0.05732 | 24.28797 | 0.00031 | 0.0022 | 0.89 | 0.047795 |
| rs76578522 | G | A | −1.97938 | 0.417324 | 2.11 × 10^−6^ | 0.015821 | 22.49633 | −0.0001 | 0.001951 | 0.96 | 0.053894 |
| rs78160994 | G | A | 1.89455 | 0.405965 | 3.06 × 10^−6^ | 0.001349 | 21.77885 | −0.00528 | 0.004193 | 0.21 | 0.011441 |
| rs78709176 | C | T | −0.78321 | 0.165384 | 2.18 × 10^−6^ | 0.04841 | 22.4268 | 1.02 × 10^−6^ | 0.002519 | 1 | 0.031347 |
| rs9597498 | A | G | −0.28285 | 0.061806 | 4.73 × 10^−6^ | 0.339862 | 20.9435 | −0.00134 | 0.000928 | 0.15 | 0.346353 |

EA: Effect allele; OA: Other allele; EAF: Effect allele frequency; SNP: Single nucleotide polymorphism; SE: Standard Error.

Supplementary Table 16. Detail information of selected SNPs for MR analysis of the causal effect of bruxism on facial pain.

| SNP ID | EA | OA | Exposure | | | | | Outcome | | | |
| --- | --- | --- | --- | --- | --- | --- | --- | --- | --- | --- | --- |
|  |  |  | Beta | SE | *p*-value | EAF | *F* | Beta | SE | *p*-value | EAF |
| rs117019503 | T | A | 0.323912 | 0.068172 | 2.02 × 10^−6^ | 0.195205 | 22.57601 | −0.00012 | 0.000431 | 0.780001 | 0.122385 |
| rs1750430 | C | T | 0.436331 | 0.090354 | 1.37 × 10^−6^ | 0.085729 | 23.32042 | −4.95 × 10^−5^ | 0.000444 | 0.91 | 0.116128 |
| rs5765490 | C | T | −0.28217 | 0.056989 | 7.37 × 10^−7^ | 0.586109 | 24.51509 | 0.000212 | 0.000288 | 0.46 | 0.607931 |
| rs76371754 | T | C | −0.77514 | 0.157284 | 8.30 × 10^−7^ | 0.05732 | 24.28797 | 0.000804 | 0.000706 | 0.26 | 0.047795 |
| rs76578522 | G | A | −1.97938 | 0.417324 | 2.11 × 10^−6^ | 0.015821 | 22.49633 | 0.000727 | 0.000627 | 0.25 | 0.053894 |
| rs9597498 | A | G | −0.28285 | 0.061806 | 4.73 × 10^−6^ | 0.339862 | 20.9435 | 0.000286 | 0.000298 | 0.34 | 0.346353 |

EA: Effect allele; OA: Other allele; EAF: Effect allele frequency; SNP: Single nucleotide polymorphism; SE: Standard Error.

Supplementary Table 17. Detail information of selected SNPs for MR analysis of the causal effect of bruxism on cluster headache.

| SNP | EA | OA | Exposure | | | | | Outcome | | | |  |
| --- | --- | --- | --- | --- | --- | --- | --- | --- | --- | --- | --- | --- |
|  |  |  | Beta | SE | *p*-value | EAF | *F* | Beta | SE | *p*-value | EAF | |
| rs117019503 | T | A | 0.323912 | 0.068172 | 2.02 × 10^−6^ | 0.195205 | 22.57601 | −0.00414 | 0.049434 | 0.93332 | 0.195065 | |
| rs139685925 | A | G | 0.501149 | 0.105944 | 2.24 × 10^−6^ | 0.057895 | 22.37593 | −0.02019 | 0.083514 | 0.808967 | 0.057982 | |
| rs144481227 | A | G | 0.836264 | 0.179232 | 3.07 × 10^−6^ | 0.014637 | 21.76986 | −0.19838 | 0.164677 | 0.228339 | 0.014606 | |
| rs147876377 | G | A | −0.78279 | 0.167925 | 3.14 × 10^−6^ | 0.046668 | 21.72989 | 0.046965 | 0.09046 | 0.603637 | 0.046605 | |
| rs1750430 | C | T | 0.436331 | 0.090354 | 1.37 × 10^−6^ | 0.085729 | 23.32042 | 0.004815 | 0.069605 | 0.94485 | 0.085473 | |
| rs5765490 | C | T | −0.28217 | 0.056989 | 7.37 × 10^−7^ | 0.586109 | 24.51509 | −0.02086 | 0.039437 | 0.59683 | 0.586348 | |
| rs76371754 | T | C | −0.77514 | 0.157284 | 8.30 × 10^−7^ | 0.05732 | 24.28797 | −0.00703 | 0.089202 | 0.937166 | 0.057407 | |
| rs76578522 | G | A | −1.97938 | 0.417324 | 2.11 × 10^−6^ | 0.015821 | 22.49633 | 0.007668 | 0.159202 | 0.961582 | 0.01583 | |
| rs78160994 | G | A | 1.89455 | 0.405965 | 3.06 × 10^−6^ | 0.001349 | 21.77885 | 0.778974 | 0.584697 | 0.182772 | 0.001342 | |
| rs78709176 | C | T | −0.78321 | 0.165384 | 2.18 × 10^−6^ | 0.04841 | 22.4268 | −0.01136 | 0.090826 | 0.900494 | 0.048153 | |
| rs9597498 | A | G | −0.28285 | 0.061806 | 4.73 × 10^−6^ | 0.339862 | 20.9435 | −0.04575 | 0.040935 | 0.263696 | 0.34009 | |

EA: Effect allele; OA: Other allele; EAF: Effect allele frequency; SNP: Single nucleotide polymorphism; SE: Standard Error.

Supplementary Table 18. Detail information of selected SNPs for MR analysis of the causal effect of bruxism on tension-types headache.

| SNP | EA | OA | Exposure | | | | | Outcome | | | |  |
| --- | --- | --- | --- | --- | --- | --- | --- | --- | --- | --- | --- | --- |
|  |  |  | Beta | SE | *p-*value | EAF | *F* | Beta | SE | *p-*value | EAF | |
| rs139685925 | A | G | 0.501149 | 0.105944 | 2.24 × 10^−6^ | 0.057895 | 22.37593 | 0.455 | 0.3 | 0.129 | 0.019943 | |
| rs144481227 | A | G | 0.836264 | 0.179232 | 3.07 × 10^−6^ | 0.014637 | 21.76986 | −0.137 | 0.505 | 0.787 | 0.007626 | |
| rs147876377 | G | A | −0.78279 | 0.167925 | 3.14 × 10^−6^ | 0.046668 | 21.72989 | −1.31 | 2.9 | 0.652 | 0.000239 | |
| rs1750430 | C | T | 0.436331 | 0.090354 | 1.37 × 10^−6^ | 0.085729 | 23.32042 | 0.123 | 0.127 | 0.332 | 0.114347 | |
| rs5765490 | C | T | −0.28217 | 0.056989 | 7.37 × 10^−7^ | 0.586109 | 24.51509 | −0.0422 | 0.0818 | 0.606 | 0.609388 | |
| rs76371754 | T | C | −0.77514 | 0.157284 | 8.30 × 10^−7^ | 0.05732 | 24.28797 | 0.22 | 0.205 | 0.284 | 0.046199 | |
| rs76578522 | G | A | −1.97938 | 0.417324 | 2.11 × 10^−6^ | 0.015821 | 22.49633 | 0.125 | 0.178 | 0.483 | 0.053769 | |
| rs78160994 | G | A | 1.89455 | 0.405965 | 3.06 × 10^−6^ | 0.001349 | 21.77885 | 0.921 | 0.378 | 0.0148 | 0.011794 | |
| rs78709176 | C | T | −0.78321 | 0.165384 | 2.18 × 10^−6^ | 0.04841 | 22.4268 | 0.163 | 0.229 | 0.477 | 0.031193 | |
| rs9597498 | A | G | −0.28285 | 0.061806 | 4.73 × 10^−6^ | 0.339862 | 20.9435 | −0.0363 | 0.0846 | 0.668 | 0.344859 | |

EA: Effect allele; OA: Other allele; EAF: Effect allele frequency; SNP: Single nucleotide polymorphism; SE: Standard Error.

Supplementary Table 19. Detail information of selected SNPs for MR analysis of the causal effect of bruxism on migraine.

| SNP ID | EA | OA | Exposure | | | | | Outcome | | | |
| --- | --- | --- | --- | --- | --- | --- | --- | --- | --- | --- | --- |
|  |  |  | Beta | SE | *p*-value | EAF | *F* | Beta | SE | *p*-value | EAF |
| rs117019503 | T | A | 0.323912 | 0.068172 | 2.02 × 10^−6^ | 0.195205 | 22.57601 | 0.000273 | 0.000929 | 0.77 | 0.04922 |
| rs139685925 | A | G | 0.501149 | 0.105944 | 2.24 × 10^−6^ | 0.057895 | 22.37593 | −0.0009 | 0.001265 | 0.51 | 0.020505 |
| rs1750430 | C | T | 0.436331 | 0.090354 | 1.37 × 10^−6^ | 0.085729 | 23.32042 | −0.00069 | 0.00053 | 0.19 | 0.126366 |
| rs5765490 | C | T | −0.28217 | 0.056989 | 7.37 × 10^−7^ | 0.586109 | 24.51509 | 0.000848 | 0.000351 | 0.015 | 0.613736 |
| rs76371754 | T | C | −0.77514 | 0.157284 | 8.30 × 10^−7^ | 0.05732 | 24.28797 | 0.000272 | 0.000837 | 0.7 | 0.050205 |
| rs76578522 | G | A | −1.97938 | 0.417324 | 2.11 × 10^−6^ | 0.015821 | 22.49633 | −0.00024 | 0.000759 | 0.73 | 0.053765 |
| rs78160994 | G | A | 1.89455 | 0.405965 | 3.06 × 10^−6^ | 0.001349 | 21.77885 | 0.00222 | 0.001658 | 0.17 | 0.011044 |
| rs78709176 | C | T | −0.78321 | 0.165384 | 2.18 × 10^−6^ | 0.04841 | 22.4268 | −0.00095 | 0.00097 | 0.31 | 0.031969 |
| rs9597498 | A | G | −0.28285 | 0.061806 | 4.73 × 10^−6^ | 0.339862 | 20.9435 | −0.00039 | 0.000364 | 0.27 | 0.339024 |

EA: Effect allele; OA: Other allele; EAF: Effect allele frequency; SNP: Single nucleotide polymorphism; SE: Standard Error.

Supplementary Table 20. Detail information of selected SNPs for MR analysis of the causal effect of headache on bruxism.

| SNP | EA | OA | Exposure | | | | | Outcome | | | |
| --- | --- | --- | --- | --- | --- | --- | --- | --- | --- | --- | --- |
|  |  |  | Beta | SE | *p*-value | EAF | *F* | Beta | SE | *p*-value | EAF |
| rs115573881 | C | T | 0.002161 | 0.0004 | 6.40 × 10^−8^ | 0.060417 | 29.23329 | −0.00145 | 0.11655 | 0.990095 | 0.068363 |
| rs140996669 | A | G | 0.003324 | 0.000667 | 6.20 × 10^−7^ | 0.021819 | 24.83482 | −0.2723 | 0.266223 | 0.306393 | 0.012874 |
| rs73393137 | T | G | −0.00145 | 0.000315 | 4.50 × 10^−6^ | 0.099976 | 21.03644 | −0.05953 | 0.132323 | 0.652776 | 0.051072 |
| rs7543791 | G | T | 0.002226 | 0.000454 | 9.40 × 10^−7^ | 0.048856 | 24.05493 | −0.03942 | 0.098035 | 0.6876 | 0.100096 |
| rs79344266 | T | G | 0.002563 | 0.000525 | 1.10 × 10^−6^ | 0.033197 | 23.80103 | −0.08828 | 0.281915 | 0.75418 | 0.010691 |
| rs9486719 | A | G | 0.001094 | 0.000235 | 3.20 × 10^−6^ | 0.200405 | 21.66523 | −0.06331 | 0.087917 | 0.471479 | 0.124768 |

EA: Effect allele; OA: Other allele; EAF: Effect allele frequency; SNP: Single nucleotide polymorphism; SE: Standard Error.

Supplementary Table 21. Detail information of selected SNPs for MR analysis of the causal effect of neck/shoulder pain on bruxism.

| SNP | EA | OA | Exposure | | | | | Outcome | | | |  |
| --- | --- | --- | --- | --- | --- | --- | --- | --- | --- | --- | --- | --- |
|  |  |  | Beta | SE | *p*-value | EAF | *F* | Beta | SE | *p*-value | EAF | |
| rs10135643 | C | A | 0.004738 | 0.000907 | 1.80 × 10^−7^ | 0.372296 | 27.27106 | −0.12802 | 0.061557 | 0.037561 | 0.345703 | |
| rs1018503 | G | T | −0.00415 | 0.000884 | 2.70 × 10^−6^ | 0.429536 | 22.02984 | 0.012417 | 0.058817 | 0.832803 | 0.444889 | |
| rs10789929 | T | C | 0.004763 | 0.000902 | 1.30 × 10^−7^ | 0.38504 | 27.8792 | 0.100148 | 0.061264 | 0.102114 | 0.628771 | |
| rs10818936 | G | C | 0.004772 | 0.000942 | 4.10 × 10^−7^ | 0.320277 | 25.66898 | −0.04655 | 0.062437 | 0.455933 | 0.326882 | |
| rs11171739 | T | C | 0.004062 | 0.000883 | 4.30 × 10^−6^ | 0.568548 | 21.14205 | −0.12596 | 0.059182 | 0.033305 | 0.570828 | |
| rs112039693 | C | T | 0.015601 | 0.003236 | 1.40 × 10^−6^ | 0.019444 | 23.23791 | 0.131082 | 0.313229 | 0.675591 | 0.00914 | |
| rs112292112 | A | G | −0.01264 | 0.002552 | 7.30 × 10^−7^ | 0.030415 | 24.52247 | 0.007958 | 0.226491 | 0.971972 | 0.015828 | |
| rs11252332 | A | T | −0.00432 | 0.000914 | 2.30 × 10^−6^ | 0.357903 | 22.32759 | 0.008055 | 0.063708 | 0.899389 | 0.301556 | |
| rs11736161 | A | G | 0.004329 | 0.000914 | 2.20 × 10^−6^ | 0.640942 | 22.41923 | −0.04183 | 0.06217 | 0.501043 | 0.66741 | |
| rs12659211 | A | T | 0.005086 | 0.001071 | 2.00 × 10^−6^ | 0.213274 | 22.56073 | 0.068052 | 0.06429 | 0.289822 | 0.295312 | |
| rs12732083 | T | C | 0.00524 | 0.001047 | 5.60 × 10^−7^ | 0.226532 | 25.06166 | −0.01054 | 0.069596 | 0.879607 | 0.231319 | |
| rs1286231 | C | T | −0.00423 | 0.000896 | 2.30 × 10^−6^ | 0.588408 | 22.2936 | 0.114807 | 0.059213 | 0.052517 | 0.583689 | |
| rs13031851 | C | T | −0.00459 | 0.0009 | 3.50 × 10^−7^ | 0.380864 | 25.93832 | −0.01945 | 0.061198 | 0.750656 | 0.351981 | |
| rs13107325 | T | C | 0.010907 | 0.001665 | 5.70 × 10^−11^ | 0.074888 | 42.93217 | 0.625657 | 0.251356 | 0.012806 | 0.013973 | |
| rs1338034 | A | G | 0.004238 | 0.000899 | 2.50 × 10^−6^ | 0.602822 | 22.2038 | −0.06761 | 0.060451 | 0.263407 | 0.627435 | |
| rs1399629 | A | G | −0.00445 | 0.000874 | 3.70 × 10^−7^ | 0.507288 | 25.84172 | −0.02312 | 0.058472 | 0.692529 | 0.480493 | |
| rs1476535 | T | C | 0.00489 | 0.000888 | 3.70 × 10^−8^ | 0.561666 | 30.32335 | −0.00976 | 0.058771 | 0.86809 | 0.524142 | |
| rs1532204 | G | C | −0.00587 | 0.001193 | 8.70 × 10^−7^ | 0.839533 | 24.1971 | −0.04048 | 0.09602 | 0.673321 | 0.893857 | |
| rs159963 | A | C | −0.00437 | 0.000891 | 9.50 × 10^−7^ | 0.581595 | 24.02026 | −0.04206 | 0.061745 | 0.495778 | 0.657081 | |
| rs16980973 | T | A | −0.00693 | 0.00132 | 1.50 × 10^−7^ | 0.126644 | 27.53774 | −0.03316 | 0.078078 | 0.67105 | 0.168143 | |
| rs1962104 | C | T | 0.004122 | 0.000889 | 3.50 × 10^−6^ | 0.558211 | 21.49408 | 0.077756 | 0.058478 | 0.183628 | 0.475639 | |
| rs1963206 | A | G | −0.00538 | 0.001064 | 4.30 × 10^−7^ | 0.782775 | 25.55661 | −0.07735 | 0.066931 | 0.2478 | 0.746937 | |
| rs198230 | C | A | −0.005 | 0.001054 | 2.10 × 10^−6^ | 0.777437 | 22.52346 | −0.09661 | 0.062637 | 0.122978 | 0.676846 | |
| rs2149814 | C | A | 0.006626 | 0.001362 | 1.10 × 10^−6^ | 0.118568 | 23.67038 | −0.06078 | 0.100308 | 0.544545 | 0.091936 | |
| rs2283076 | G | A | 0.005173 | 0.001045 | 7.50 × 10^−7^ | 0.22942 | 24.49265 | −0.00438 | 0.081575 | 0.957174 | 0.152965 | |
| rs2587363 | A | G | −0.00443 | 0.000885 | 5.40 × 10^−7^ | 0.569798 | 25.12913 | −0.02127 | 0.058713 | 0.717196 | 0.566869 | |
| rs270833 | G | A | −0.00412 | 0.000877 | 2.60 × 10^−6^ | 0.47098 | 22.09104 | 0.048829 | 0.058635 | 0.404975 | 0.537646 | |
| rs274622 | T | C | −0.00426 | 0.00092 | 3.70 × 10^−6^ | 0.652806 | 21.40897 | 0.01698 | 0.059871 | 0.776714 | 0.604957 | |
| rs2898518 | A | G | 0.004078 | 0.000891 | 4.70 × 10^−6^ | 0.413944 | 20.96293 | 0.035598 | 0.060073 | 0.55346 | 0.379264 | |
| rs2919935 | T | A | 0.007877 | 0.001642 | 1.60 × 10^−6^ | 0.077051 | 23.0207 | −0.08429 | 0.099194 | 0.395466 | 0.096527 | |
| rs3132469 | G | A | 0.005705 | 0.001227 | 3.30 × 10^−6^ | 0.850998 | 21.63405 | −0.06043 | 0.085397 | 0.479133 | 0.868778 | |
| rs34332831 | T | C | 0.005923 | 0.001283 | 3.90 × 10^−6^ | 0.134811 | 21.32493 | −0.0707 | 0.086715 | 0.414921 | 0.127993 | |
| rs35919525 | G | T | 0.008331 | 0.001775 | 2.70 × 10^−6^ | 0.066366 | 22.04156 | 0.075206 | 0.104449 | 0.47151 | 0.086999 | |
| rs36042757 | C | A | −0.00687 | 0.001274 | 7.00 × 10^−8^ | 0.137385 | 29.05402 | −0.06022 | 0.100884 | 0.550548 | 0.091371 | |
| rs4141466 | C | T | 0.004447 | 0.000937 | 2.10 × 10^−6^ | 0.327824 | 22.54145 | 0.017712 | 0.060365 | 0.769207 | 0.368943 | |
| rs4518923 | G | A | −0.00473 | 0.000929 | 3.50 × 10^−7^ | 0.350504 | 25.93378 | −0.04894 | 0.059589 | 0.411523 | 0.413316 | |
| rs4886860 | C | G | −0.00548 | 0.001035 | 1.20 × 10^−7^ | 0.767425 | 28.00519 | −0.01842 | 0.063633 | 0.772209 | 0.69878 | |
| rs55670730 | T | A | 0.0066 | 0.00141 | 2.90 × 10^−6^ | 0.110749 | 21.91179 | −0.1035 | 0.093125 | 0.266385 | 0.109676 | |
| rs55886108 | T | C | −0.00415 | 0.0009 | 3.90 × 10^−6^ | 0.388443 | 21.31086 | −0.0699 | 0.061891 | 0.258721 | 0.337482 | |
| rs56069701 | T | C | −0.00726 | 0.001542 | 2.50 × 10^−6^ | 0.093227 | 22.16774 | −0.39209 | 0.154189 | 0.010994 | 0.03667 | |
| rs568809 | G | C | 0.00549 | 0.001119 | 9.20 × 10^−7^ | 0.808201 | 24.08356 | 0.050406 | 0.08969 | 0.574115 | 0.877883 | |
| rs58621819 | T | A | 0.005192 | 0.001064 | 1.10 × 10^−6^ | 0.217556 | 23.83324 | 0.191598 | 0.104088 | 0.065661 | 0.089787 | |
| rs6041749 | C | T | 0.004556 | 0.000932 | 1.00 × 10^−6^ | 0.440756 | 23.91464 | 0.060333 | 0.063938 | 0.345367 | 0.323693 | |
| rs6062964 | T | C | −0.02085 | 0.00409 | 3.50 × 10^−7^ | 0.011778 | 25.97624 | −0.37921 | 0.301765 | 0.208878 | 0.009488 | |
| rs6119944 | G | A | 0.005294 | 0.001041 | 3.60 × 10^−7^ | 0.239843 | 25.88159 | −0.01882 | 0.063245 | 0.765974 | 0.313566 | |
| rs61779314 | T | C | 0.006177 | 0.001128 | 4.30 × 10^−8^ | 0.185979 | 30.00269 | −0.03757 | 0.071937 | 0.601532 | 0.203573 | |
| rs62056490 | A | C | −0.0062 | 0.001089 | 1.20 × 10^−8^ | 0.794202 | 32.45511 | −0.01067 | 0.08107 | 0.895302 | 0.842664 | |
| rs62131354 | T | C | 0.004821 | 0.001048 | 4.20 × 10^−6^ | 0.2257 | 21.16933 | 0.106484 | 0.063629 | 0.094224 | 0.304143 | |
| rs6606710 | C | T | 0.004566 | 0.000922 | 7.40 × 10^−7^ | 0.406931 | 24.51218 | 0.101394 | 0.06283 | 0.106573 | 0.331941 | |
| rs6935368 | T | C | −0.00454 | 0.000904 | 5.10 × 10^−7^ | 0.380828 | 25.23667 | −0.01797 | 0.059305 | 0.761861 | 0.429287 | |
| rs71607173 | G | C | 0.016426 | 0.003533 | 3.30 × 10^−6^ | 0.016225 | 21.61591 | 0.324506 | 0.236638 | 0.170276 | 0.015001 | |
| rs7232237 | G | A | 0.004682 | 0.000875 | 8.80 × 10^−8^ | 0.501939 | 28.62137 | −0.107 | 0.058685 | 0.068268 | 0.539833 | |
| rs7248205 | T | C | −0.00502 | 0.000896 | 2.10 × 10^−8^ | 0.600262 | 31.42091 | −0.00263 | 0.05976 | 0.964918 | 0.607852 | |
| rs72671456 | A | G | 0.006586 | 0.001282 | 2.80 × 10^−7^ | 0.135444 | 26.39565 | −0.08048 | 0.099435 | 0.418296 | 0.095991 | |
| rs729053 | C | G | 0.005082 | 0.000932 | 5.00 × 10^−8^ | 0.332118 | 29.71121 | −0.03938 | 0.063204 | 0.533213 | 0.312317 | |
| rs7297794 | T | C | −0.00512 | 0.001052 | 1.10 × 10^−6^ | 0.224021 | 23.66614 | −0.00442 | 0.066259 | 0.946822 | 0.265307 | |
| rs7317962 | T | G | 0.004724 | 0.000895 | 1.30 × 10^−7^ | 0.605114 | 27.85487 | 0.003539 | 0.058922 | 0.952113 | 0.52964 | |
| rs7739896 | G | A | −0.00418 | 0.000878 | 1.90 × 10^−6^ | 0.531089 | 22.6909 | −0.06616 | 0.058353 | 0.256866 | 0.496257 | |
| rs78169991 | G | A | −0.01454 | 0.002722 | 9.10 × 10^−8^ | 0.027472 | 28.55927 | −0.14 | 0.167853 | 0.404265 | 0.030815 | |
| rs8054093 | C | A | −0.00431 | 0.000892 | 1.30 × 10^−6^ | 0.563325 | 23.41735 | −0.04327 | 0.061338 | 0.480537 | 0.643785 | |
| rs9375610 | G | A | 0.004078 | 0.000888 | 4.40 × 10^−6^ | 0.580922 | 21.07859 | 0.015438 | 0.060358 | 0.798127 | 0.619566 | |
| rs9889282 | C | A | 0.006232 | 0.000902 | 4.80 × 10^−12^ | 0.387918 | 47.75088 | −0.02069 | 0.060554 | 0.732543 | 0.371767 | |

EA: Effect allele; OA: Other allele; EAF: Effect allele frequency; SNP: Single nucleotide polymorphism; SE: Standard Error.

Supplementary Table 22. Detail information of selected SNPs for MR analysis of the causal effect of facial pain on bruxism.

| SNP | EA | OA | Exposure | | | | | Outcome | | | |
| --- | --- | --- | --- | --- | --- | --- | --- | --- | --- | --- | --- |
|  |  |  | Beta | SE | *p*-value | EAF | *F* | Beta | SE | *p*-value | EAF |
| rs12445269 | T | C | −0.00147 | 0.000286 | 2.90 × 10^−7^ | 0.41534 | 26.33085 | −0.0479 | 0.060645 | 0.429614 | 0.370125 |
| rs12712737 | G | A | 0.001344 | 0.000287 | 2.70 × 10^−6^ | 0.420497 | 22.00116 | 0.060507 | 0.059636 | 0.310291 | 0.400524 |
| rs2199214 | C | T | 0.001719 | 0.000374 | 4.40 × 10^−6^ | 0.172202 | 21.07934 | −0.03673 | 0.080089 | 0.646532 | 0.16244 |
| rs34234617 | G | C | −0.00305 | 0.000648 | 2.50 × 10^−6^ | 0.051931 | 22.20504 | 0.19117 | 0.118057 | 0.105384 | 0.065899 |
| rs34311004 | C | T | 0.003352 | 0.000711 | 2.40 × 10^−6^ | 0.042334 | 22.24341 | −0.01596 | 0.184074 | 0.930905 | 0.025755 |
| rs4416176 | C | T | 0.002218 | 0.000477 | 3.30 × 10^−6^ | 0.095787 | 21.62604 | −0.00114 | 0.113366 | 0.991952 | 0.073499 |
| rs6967391 | T | A | 0.001753 | 0.00036 | 1.10 × 10^−6^ | 0.195562 | 23.67744 | 0.042763 | 0.067484 | 0.526292 | 0.257158 |
| rs72694537 | T | C | 0.00164 | 0.000355 | 3.80 × 10^−6^ | 0.199463 | 21.34035 | −0.11867 | 0.076824 | 0.122433 | 0.174893 |
| rs72711207 | C | T | −0.00228 | 0.000476 | 1.70 × 10^−6^ | 0.0976 | 22.95158 | −0.05386 | 0.114486 | 0.638052 | 0.071827 |
| rs76714322 | T | C | 0.002189 | 0.000458 | 1.70 × 10^−6^ | 0.105959 | 22.87755 | 0.051478 | 0.085311 | 0.546228 | 0.13517 |
| rs7937459 | A | C | 0.001399 | 0.000291 | 1.60 × 10^−6^ | 0.402997 | 23.05739 | 0.043246 | 0.058993 | 0.463511 | 0.458535 |
| rs7965652 | A | G | 0.001379 | 0.000293 | 2.60 × 10^−6^ | 0.354152 | 22.09799 | −0.13044 | 0.065504 | 0.046452 | 0.278167 |
| rs8003983 | A | C | −0.00193 | 0.000407 | 2.20 × 10^−6^ | 0.1504 | 22.3747 | −0.06567 | 0.085044 | 0.439971 | 0.14103 |
| rs8005612 | C | T | −0.00166 | 0.000349 | 1.90 × 10^−6^ | 0.207409 | 22.69808 | 0.182351 | 0.072662 | 0.012088 | 0.200206 |
| rs9643685 | T | C | −0.00153 | 0.000333 | 4.30 × 10^−6^ | 0.765689 | 21.12775 | 0.101623 | 0.078951 | 0.198039 | 0.839465 |
| rs9645327 | T | C | 0.002054 | 0.00044 | 3.00 × 10^−6^ | 0.117246 | 21.82874 | −0.00342 | 0.086333 | 0.968395 | 0.129206 |

EA: Effect allele; OA: Other allele; EAF: Effect allele frequency; SNP: Single nucleotide polymorphism; SE: Standard Error.

Supplementary Table 23. Detail information of selected SNPs for MR analysis of the causal effect of cluster headache on bruxism.

| SNP | EA | OA | Exposure | | | | | Outcome | | | |
| --- | --- | --- | --- | --- | --- | --- | --- | --- | --- | --- | --- |
|  |  |  | Beta | SE | *p*-value | EAF | *F* | Beta | SE | *p*-value | EAF |
| rs111389066 | C | G | −1.12758 | 0.242603 | 3.35 × 10^−6^ | 0.012873 | 21.60242 | −0.47886 | 0.260978 | 0.066524 | 0.013023 |
| rs113464711 | CTTATTCA | C | −0.17656 | 0.038238 | 3.88 × 10^−6^ | 0.531978 | 21.32139 | −0.08648 | 0.058605 | 0.140042 | 0.531912 |
| rs118067737 | T | A | −1.33881 | 0.28874 | 3.54 × 10^−6^ | 0.01003 | 21.49928 | −0.12878 | 0.299281 | 0.66697 | 0.009875 |
| rs118091971 | A | G | 0.520692 | 0.112553 | 3.72 × 10^−6^ | 0.022599 | 21.40167 | −0.03164 | 0.200353 | 0.874528 | 0.022457 |
| rs141034388 | T | C | 0.523304 | 0.108484 | 1.41 × 10^−6^ | 0.023735 | 23.26895 | −0.19683 | 0.200281 | 0.325732 | 0.023526 |
| rs185426866 | T | A | 0.593014 | 0.121285 | 1.01 × 10^−6^ | 0.018514 | 23.90648 | −0.09416 | 0.217924 | 0.665691 | 0.01853 |
| rs372466710 | C | T | −0.68963 | 0.141341 | 1.07 × 10^−6^ | 0.039839 | 23.80659 | −0.00656 | 0.180008 | 0.970923 | 0.039756 |
| rs3845509 | C | A | 0.192701 | 0.04175 | 3.92 × 10^−6^ | 0.270675 | 21.30349 | 0.042605 | 0.065721 | 0.516811 | 0.270348 |
| rs4560253 | G | C | −0.21466 | 0.042233 | 3.72 × 10^−7^ | 0.318889 | 25.83428 | 0.030314 | 0.062981 | 0.630289 | 0.31864 |
| rs6135407 | T | C | −0.26107 | 0.052567 | 6.82 × 10^−7^ | 0.178351 | 24.66489 | −0.07654 | 0.076049 | 0.314196 | 0.178599 |
| rs6508354 | C | T | 0.243459 | 0.050869 | 1.70 × 10^−6^ | 0.147766 | 22.90588 | 0.091321 | 0.082014 | 0.265503 | 0.14831 |
| rs7291345 | C | G | −0.28026 | 0.060235 | 3.28 × 10^−6^ | 0.13215 | 21.64787 | −0.13085 | 0.08558 | 0.126266 | 0.132475 |
| rs73083559 | T | C | −2.89007 | 0.605983 | 1.85 × 10^−6^ | 0.005394 | 22.74552 | −0.00398 | 0.411058 | 0.992274 | 0.005362 |
| rs73310223 | T | A | 0.579721 | 0.124217 | 3.06 × 10^−6^ | 0.017241 | 21.78091 | 0.122414 | 0.224882 | 0.586203 | 0.017303 |
| rs78350310 | T | C | 0.392327 | 0.08542 | 4.37 × 10^−6^ | 0.043127 | 21.09474 | 0.125759 | 0.145627 | 0.387826 | 0.042911 |

EA: Effect allele; OA: Other allele; EAF: Effect allele frequency; SNP: Single nucleotide polymorphism; SE: Standard Error.

Supplementary Table 24. Detail information of selected SNPs for MR analysis of the causal effect of tension-types headache on bruxism.

| SNP | EA | OA | Exposure | | | | | Outcome | | | |
| --- | --- | --- | --- | --- | --- | --- | --- | --- | --- | --- | --- |
|  |  |  | Beta | SE | *p*-value | EAF | *F* | Beta | SE | *p*-value | EAF |
| rs12349132 | T | C | −0.13113 | 0.466 | 4.80 × 10^−6^ | 0.010762 | 20.89235 | 0.010762 | 0.662812 | 0.843168 | 0.002116 |
| rs139386387 | A | G | 0.462275 | 0.287 | 4.38 × 10^−6^ | 0.02713 | 21.15359 | 0.02713 | 0.27464 | 0.092336 | 0.011902 |
| rs140562886 | T | C | −0.01431 | 0.404 | 4.74 × 10^−6^ | 0.014439 | 20.96915 | 0.014439 | 0.579023 | 0.980279 | 0.002623 |
| rs28699426 | A | T | 0.03958 | 0.09 | 3.99 × 10^−6^ | 0.282978 | 21.26235 | 0.282978 | 0.068646 | 0.564225 | 0.24563 |
| rs3810037 | T | C | 0.031733 | 0.091 | 7.59 × 10^−7^ | 0.278832 | 24.45357 | 0.278832 | 0.064258 | 0.621423 | 0.295448 |
| rs4784924 | T | G | −0.08734 | 0.0833 | 1.48 × 10^−6^ | 0.625173 | 23.17388 | 0.625173 | 0.060129 | 0.146329 | 0.610794 |
| rs58692695 | A | G | 0.101305 | 0.133 | 7.14 × 10^−7^ | 0.115647 | 24.77494 | 0.115647 | 0.121536 | 0.404544 | 0.06197 |
| rs61992090 | C | T | −0.48715 | 0.335 | 1.06 × 10^−6^ | 0.019763 | 23.67476 | 0.019763 | 0.420739 | 0.246926 | 0.005289 |

EA: Effect allele; OA: Other allele; EAF: Effect allele frequency; SNP: Single nucleotide polymorphism; SE: Standard Error.

Supplementary Table 25. Detail information of selected SNPs for MR analysis of the causal effect of migraine on bruxism.

| SNP | EA | OA | Exposure | | | | | Outcome | | | |  |
| --- | --- | --- | --- | --- | --- | --- | --- | --- | --- | --- | --- | --- |
|  |  |  | Beta | SE | *p*-value | EAF | *F* | Beta | SE | *p*-value | EAF | |
| rs10023050 | G | A | −0.00167 | 0.000349 | 1.40 × 10^−6^ | 0.39164 | 22.84381 | 0.087006 | 0.058692 | 0.13823 | 0.467942 | |
| rs10127740 | T | G | −0.00169 | 0.000371 | 4.40 × 10^−6^ | 0.310689 | 20.7358 | −0.00362 | 0.065079 | 0.955587 | 0.280027 | |
| rs10218452 | G | A | 0.004788 | 0.000407 | 7.40 × 10^−32^ | 0.229834 | 138.5081 | 0.011413 | 0.067945 | 0.866602 | 0.245184 | |
| rs1047891 | A | C | 0.0017 | 0.000365 | 3.20 × 10^−6^ | 0.315455 | 21.6511 | −0.04311 | 0.062385 | 0.489587 | 0.32225 | |
| rs10927722 | A | G | 0.001814 | 0.000356 | 3.20 × 10^−7^ | 0.365356 | 25.99966 | −0.05033 | 0.064951 | 0.438439 | 0.282408 | |
| rs11153082 | G | A | 0.003417 | 0.000364 | 5.20 × 10^−21^ | 0.325011 | 88.30689 | −0.06307 | 0.063616 | 0.32151 | 0.298545 | |
| rs11172113 | C | T | −0.00392 | 0.000346 | 7.60 × 10^−30^ | 0.411057 | 128.6477 | 0.016873 | 0.05958 | 0.777025 | 0.399436 | |
| rs11602707 | T | C | 0.001906 | 0.000401 | 2.10 × 10^−6^ | 0.238508 | 22.56948 | 0.128564 | 0.066644 | 0.053717 | 0.258574 | |
| rs11853918 | T | C | 0.001781 | 0.000381 | 2.70 × 10^−6^ | 0.278792 | 21.83689 | −0.08446 | 0.065352 | 0.196236 | 0.277785 | |
| rs12070846 | C | T | 0.00208 | 0.000409 | 3.40 × 10^−7^ | 0.227741 | 25.83472 | 0.100535 | 0.075594 | 0.183541 | 0.186875 | |
| rs12134493 | A | C | 0.004167 | 0.000533 | 7.10 × 10^−15^ | 0.116843 | 61.14325 | −0.01101 | 0.085216 | 0.897212 | 0.137106 | |
| rs12295710 | T | C | 0.002062 | 0.000342 | 2.00 × 10^−9^ | 0.46249 | 36.26075 | −0.03009 | 0.058364 | 0.60615 | 0.47823 | |
| rs12452590 | G | T | 0.001731 | 0.000361 | 1.30 × 10^−6^ | 0.357283 | 23.04152 | 0.08485 | 0.059208 | 0.151835 | 0.425037 | |
| rs12684144 | C | T | 0.003524 | 0.000406 | 4.30 × 10^−18^ | 0.227875 | 75.43799 | 0.021 | 0.071287 | 0.768318 | 0.213686 | |
| rs12789511 | A | G | 0.001943 | 0.000382 | 3.30 × 10^−7^ | 0.305081 | 25.91045 | 0.146564 | 0.065003 | 0.02415 | 0.277694 | |
| rs12882067 | A | C | −0.00165 | 0.000358 | 4.80 × 10^−6^ | 0.644006 | 21.19932 | 0.12196 | 0.058674 | 0.037655 | 0.543832 | |
| rs12898361 | T | C | 0.001682 | 0.000346 | 1.10 × 10^−6^ | 0.552977 | 23.68774 | 0.113787 | 0.059575 | 0.056135 | 0.583381 | |
| rs12903810 | T | C | 0.002077 | 0.000401 | 3.00 × 10^−7^ | 0.235965 | 26.75729 | −0.01858 | 0.063957 | 0.771418 | 0.292154 | |
| rs13078967 | C | A | −0.00569 | 0.001139 | 7.10 × 10^−7^ | 0.023504 | 24.94672 | −0.42517 | 0.143555 | 0.003059 | 0.051935 | |
| rs149844910 | T | A | 0.004574 | 0.000982 | 3.30 × 10^−6^ | 0.032232 | 21.69751 | 0.049354 | 0.134925 | 0.714523 | 0.048412 | |
| rs16914944 | T | C | −0.00213 | 0.000394 | 5.70 × 10^−8^ | 0.250015 | 29.27176 | −0.02984 | 0.0645 | 0.643664 | 0.28821 | |
| rs17298647 | C | G | 0.002729 | 0.000531 | 2.50 × 10^−7^ | 0.116055 | 26.4234 | −0.04217 | 0.106658 | 0.692575 | 0.081209 | |
| rs181141583 | C | T | −0.0021 | 0.000438 | 1.40 × 10^−6^ | 0.192938 | 22.9744 | 0.143719 | 0.084864 | 0.090353 | 0.13877 | |
| rs2072151 | C | T | 0.00233 | 0.000509 | 4.40 × 10^−6^ | 0.133732 | 20.9511 | 0.004437 | 0.084274 | 0.958016 | 0.138588 | |
| rs2080685 | G | T | 0.002278 | 0.000347 | 5.40 × 10^−11^ | 0.476456 | 43.13956 | −0.03555 | 0.058573 | 0.543865 | 0.51111 | |
| rs227890 | A | G | 0.001819 | 0.000363 | 5.20 × 10^−7^ | 0.340482 | 25.07991 | 0.012432 | 0.06476 | 0.84777 | 0.287354 | |
| rs2294898 | T | G | −0.00271 | 0.000536 | 5.10 × 10^−7^ | 0.113563 | 25.57964 | −0.02317 | 0.080514 | 0.773562 | 0.15346 | |
| rs2317130 | T | C | 0.00175 | 0.000372 | 2.80 × 10^−6^ | 0.70118 | 22.14461 | −0.06081 | 0.064941 | 0.349066 | 0.720476 | |
| rs2840979 | A | G | 0.0017 | 0.000346 | 8.40 × 10^−7^ | 0.434618 | 24.18001 | −0.03904 | 0.05848 | 0.504386 | 0.483952 | |
| rs28451064 | A | G | −0.00279 | 0.000523 | 7.40 × 10^−8^ | 0.126988 | 28.54511 | 0.041509 | 0.081135 | 0.608927 | 0.153385 | |
| rs28731773 | C | T | 0.00241 | 0.000514 | 2.90 × 10^−6^ | 0.12801 | 21.97436 | 0.210291 | 0.119809 | 0.079221 | 0.065445 | |
| rs28929474 | T | C | 0.006168 | 0.001249 | 8.00 × 10^−7^ | 0.01893 | 24.39508 | −0.09799 | 0.210054 | 0.640873 | 0.019777 | |
| rs2905065 | T | C | −0.00185 | 0.000362 | 4.20 × 10^−7^ | 0.668718 | 26.033 | −0.04379 | 0.060664 | 0.470417 | 0.625982 | |
| rs34472962 | T | C | 0.004864 | 0.001061 | 4.00 × 10^−6^ | 0.029948 | 21.00345 | 0.108437 | 0.171212 | 0.526506 | 0.03073 | |
| rs35266980 | T | C | 0.002017 | 0.000371 | 5.10 × 10^−8^ | 0.310822 | 29.60544 | −0.02014 | 0.060049 | 0.737283 | 0.381648 | |
| rs4145901 | G | A | −0.00212 | 0.000406 | 1.90 × 10^−7^ | 0.769732 | 27.40681 | 0.005791 | 0.070567 | 0.934599 | 0.783096 | |
| rs4668251 | G | C | 0.001868 | 0.000376 | 5.30 × 10^−7^ | 0.694853 | 24.74388 | 0.029572 | 0.06774 | 0.662435 | 0.753687 | |
| rs4940804 | T | C | −0.0022 | 0.00042 | 1.40 × 10^−7^ | 0.206949 | 27.4537 | −0.02 | 0.067907 | 0.768401 | 0.244994 | |
| rs57943154 | A | C | 0.005364 | 0.001111 | 1.50 × 10^−6^ | 0.026322 | 23.32176 | 0.214008 | 0.228803 | 0.349613 | 0.016386 | |
| rs6088409 | G | A | 0.001846 | 0.000381 | 1.20 × 10^−6^ | 0.288645 | 23.45579 | 0.079429 | 0.064656 | 0.219263 | 0.286175 | |
| rs61747468 | A | G | 0.00613 | 0.001303 | 3.10 × 10^−6^ | 0.018096 | 22.13349 | −0.19094 | 0.200138 | 0.340061 | 0.021941 | |
| rs6432057 | A | G | −0.00174 | 0.000362 | 1.70 × 10^−6^ | 0.668861 | 23.04958 | −0.0085 | 0.063237 | 0.893105 | 0.698721 | |
| rs6700679 | T | C | −0.00251 | 0.000359 | 2.10 × 10^−12^ | 0.647038 | 49.15961 | 0.031876 | 0.059082 | 0.58953 | 0.579712 | |
| rs6738979 | G | A | −0.0033 | 0.000428 | 1.40 × 10^−14^ | 0.214035 | 59.73908 | 0.053921 | 0.080227 | 0.501519 | 0.157556 | |
| rs6815864 | C | T | 0.002223 | 0.000415 | 7.70 × 10^−8^ | 0.218767 | 28.69334 | 0.135278 | 0.074046 | 0.067706 | 0.191229 | |
| rs71558798 | G | A | 0.003437 | 0.000688 | 5.60 × 10^−7^ | 0.067766 | 24.96708 | 0.07562 | 0.134062 | 0.572708 | 0.051158 | |
| rs73138150 | T | A | 0.00188 | 0.000369 | 3.30 × 10^−7^ | 0.314968 | 25.9194 | 0.050289 | 0.061134 | 0.410735 | 0.355518 | |
| rs73196261 | G | C | −0.00774 | 0.001586 | 9.10 × 10^−7^ | 0.012061 | 23.78001 | −0.50307 | 0.579833 | 0.385604 | 0.002652 | |
| rs7757975 | T | G | 0.003328 | 0.000464 | 1.10 × 10^−12^ | 0.160036 | 51.398 | 0.169296 | 0.093848 | 0.071241 | 0.11074 | |
| rs7758604 | C | T | 0.001963 | 0.000427 | 4.00 × 10^−6^ | 0.203395 | 21.17618 | −0.0787 | 0.068935 | 0.253585 | 0.235088 | |
| rs79486579 | C | A | 0.004368 | 0.000552 | 3.40 × 10^−15^ | 0.107432 | 62.5131 | 0.076769 | 0.081869 | 0.348398 | 0.149084 | |
| rs8075138 | T | C | 0.001613 | 0.000352 | 4.60 × 10^−6^ | 0.392269 | 20.95387 | 0.023254 | 0.059889 | 0.697806 | 0.391707 | |
| rs827396 | T | C | −0.0018 | 0.000376 | 1.90 × 10^−6^ | 0.707575 | 22.83055 | −0.00254 | 0.065454 | 0.969077 | 0.726909 | |
| rs903808 | C | T | −0.00208 | 0.000356 | 5.70 × 10^−9^ | 0.635526 | 33.99467 | 0.004232 | 0.061495 | 0.945136 | 0.648725 | |
| rs914738 | C | T | −0.00184 | 0.000405 | 4.70 × 10^−6^ | 0.69359 | 20.69426 | −0.05285 | 0.061998 | 0.393941 | 0.639346 | |
| rs9349379 | G | A | −0.00319 | 0.000349 | 5.60 × 10^−20^ | 0.402367 | 83.57501 | −0.15458 | 0.05783 | 0.007518 | 0.452261 | |
| rs9847964 | T | C | 0.001811 | 0.000369 | 1.00 × 10^−6^ | 0.323769 | 24.04911 | −0.02762 | 0.061228 | 0.651885 | 0.353079 | |

EA: Effect allele; OA: Other allele; EAF: Effect allele frequency; SNP: Single nucleotide polymorphism; SE: Standard Error.
